# Supplementary material for: Osteosarcoma tumors maintain intra-tumoral transcriptional heterogeneity during bone and lung colonization
Source: BMC Biol. 2023 Apr 27;21:98. doi: 10.1186/s12915-023-01593-3 (PMC10142502; doi:10.1186/s12915-023-01593-3)
Supplement: Supplementary file 1 — Additional file 1: Table S1. Characteristics of Patient-Derived Tumor Xenograft Models used in this study. Table S2. Relative percentage of cells distributed between clusters in each model between Tibia and Lung colonization condition. Table S3. Lineage tag distribution statistics for two biological replicates. Table S4. Lineage tag summary statistics for two biological replicates. Table S5. Table showing differentially upregulated genes in the top ten enriched clonal families relative to the remaining metastatic OS-17 cells. [file 12915_2023_1593_MOESM1_ESM.docx]

Additional file 1: Supplementary Tables S1 – S5

Table S1. Characteristics of Patient-Derived Tumor Xenograft Models used in this study.

| **Module** | **Field** | **NCH-OS-2** | **NCH-OS-7** |
| --- | --- | --- | --- |
| **Clinical/patient** | Submitter patient ID | X0070 | X0140 |
|  | Gender | Male | Female |
|  | Age | 20-25 | 15-20 |
|  | Diagnosis | Metastatic osteosarcoma | Metastatic osteosarcoma |
|  | Consent to share data | Available to academic centers only | Yes |
|  | Ethnicity/race | Caucasian | Caucasian |
|  | Current treatment drug |  |  |
|  | Virology status | HepA(-), HepB(-), HepC(-), HIV-1(-), HIV-2(-), Mycoplasma(-) | HepA(-), HepB(-), HepC(-), HIV-1(-), HIV-2(-), Mycoplasma(-) |
| **Clinical/tumor** | Submitter tumor ID | X0070 | X0140 |
|  | Primary tumor tissue of origin | Bone | Bone |
|  | Primary, metastasis, recurrence | Metastasis | Recurrence |
|  | Specimen tumor tissue | Lung | Lung |
|  | Tumor grade; classification | High Grade | High grade |
|  | Disease stage; classification | metastatic osteosarcoma | metastatic osteosarcoma |
|  | Specific markers (diagnostic linked); platform | N/A | N/A |
|  | Is tumor from untreated patient? | Yes | No |
|  | Original tumor sample type | Surgical resection | Open biopsy |
|  | Tumor from an existing PDX model? ID? Why sub-line? | No | No |
| **Model creation** | Submitter PDX ID | NCH-OS-2 | NCH-OS-7 |
|  | Mouse strain (and source) | C.B-17/IcrHan Hsd-Prkdcscid | C.B-17/IcrHan Hsd-Prkdcscid |
|  | Strain immune system humanized? | No | No |
|  | Type of humanization | NA | NA |
|  | Tumor preparation | Solid tumor fragments and cell suspension | Solid tumor fragments and cell suspension |
|  | Injection type and site | Subcutaneous, flank; Intravenous, tail vien; Intraosseous, tibial plate | Subcutaneous, flank; Intravenous, tail vien; Intraosseous, tibial plate |
| **Model quality assurance** | Tumor characterization technology | Histology and IHC | Histology and IHC |
|  | Tumor confirmed not to be of mouse/EBV origin | 84% Human tissue | 76% Human tissue |
|  | Passage QA performed | Passage P3 | Passage P5 |
| **Model study** | Treatment, passage | No previous treatment | No previous treatment |
| **Associated metadata** | PDX model availability? | Yes, frozen tumor | Yes, frozen tumor |
|  | Governance restriction for distribution | Available to academic centers only | Available to academic centers only |

Table S2. Relative percentage of cells distributed between clusters in each model between Tibia and Lung colonization conditions.

| OS-17 | Condition | Cluster | Relative percentage |
| --- | --- | --- | --- |
|  | Tibia | 0 | 50.26325 |
|  | Tibia | 1 | 43.45384 |
|  | Tibia | 2 | 1.053001 |
|  | Tibia | 3 | 5.229905 |
|  | Lung | 0 | 46.57775 |
|  | Lung | 1 | 10.17901 |
|  | Lung | 2 | 39.69814 |
|  | Lung | 3 | 3.545104 |
| 143B | Tibia | 0 | 97.89638 |
|  | Tibia | 1 | 1.149201 |
|  | Tibia | 2 | 0.447994 |
|  | Tibia | 3 | 0.506428 |
|  | Lung | 0 | 2.220491 |
|  | Lung | 1 | 92.30619 |
|  | Lung | 2 | 3.350214 |
|  | Lung | 3 | 2.123101 |
| NCH-OS-2 | Tibia | 0 | 82.28155 |
|  | Tibia | 1 | 5.339806 |
|  | Tibia | 2 | 12.37864 |
|  | Lung | 0 | 20.32767 |
|  | Lung | 1 | 71.48058 |
|  | Lung | 2 | 8.191748 |
| NCH-OS-2 | Tibia | 0 | 0 |
|  | Tibia | 1 | 59.4258 |
|  | Tibia | 2 | 40.5742 |
|  | Tibia | 3 | 0 |
|  | Lung | 0 | 71.01349 |
|  | Lung | 1 | 1.383604 |
|  | Lung | 2 | 1.176064 |
|  | Lung | 3 | 26.42684 |

Table S3. Lineage tag distribution statistics for two biological replicates.

| Replicate 1 | | |
| --- | --- | --- |
| Number of copies | Frequency | Percentage |
| 1 | 2283 | 69.4 |
| 2 | 659 | 20.0 |
| 3 | 217 | 6.6 |
| 4 | 76 | 2.3 |
| 5 | 32 | 1.0 |
| 6 | 10 | 0.3 |
| 7 | 9 | 0.3 |
| 11 | 1 | 0.0 |
| 13 | 1 | 0.0 |
| 14 | 1 | 0.0 |
| Replicate 2 | | |
| Number of copies | Frequency | Percentage |
| 1 | 482 | 84.0 |
| 2 | 69 | 12.0 |
| 3 | 15 | 2.6 |
| 4 | 6 | 1.0 |
| 5 | 2 | 0.3 |

Table S4. Lineage tag summary statistics for two biological replicates.

| Replicate 1 | |
| --- | --- |
| Summary Statistics | |
| Min. | 1 |
| 1stQu. | 1 |
| Median | 1 |
| Mean | 1.483 |
| 3rdQu. | 2 |
| Max. | 14 |
| Replicate 2 | |
| Summary Statistics | |
| Min. | 1 |
| 1stQu. | 1 |
| Median | 1 |
| Mean | 1.218 |
| 3rdQu. | 1 |
| Max. | 5 |

Table S5. Table showing differentially upregulated genes in the top ten enriched clonal families relative to the remaining metastatic OS-17 cells.

|  | P value | Average log2(fold-change) | Adjusted p value |
| --- | --- | --- | --- |
| PTPRZ1 | 3.49E-24 | 0.340175 | 7.04E-20 |
| CD9 | 9.46E-21 | 0.522326 | 1.91E-16 |
| GZMB | 3.52E-20 | 0.360582 | 7.1E-16 |
| ENO2 | 8.76E-16 | 0.516978 | 1.77E-11 |
| AQP1 | 1.84E-15 | 0.298789 | 3.71E-11 |
| CDC42EP3 | 2.58E-14 | 0.302923 | 5.2E-10 |
| P4HA2 | 1.1E-12 | 0.515958 | 2.21E-08 |
| TGFBI | 1.57E-12 | 0.522179 | 3.16E-08 |
| COL6A2 | 3.66E-12 | 0.456208 | 7.37E-08 |
| BEX1 | 9.34E-12 | 0.252685 | 1.88E-07 |
| WDR54 | 1.21E-11 | 0.264337 | 2.44E-07 |
| S100A10 | 6.43E-11 | 0.308953 | 1.3E-06 |
| MIF | 7.32E-10 | 0.370393 | 1.47E-05 |
| TPI1 | 4.65E-09 | 0.285224 | 9.38E-05 |
| ENO1 | 6.68E-09 | 0.468255 | 0.000135 |
| VEGFA | 2.77E-08 | 0.752467 | 0.000558 |
| FLNB | 2.92E-08 | 0.260708 | 0.000589 |
| COL6A1 | 1.18E-07 | 0.287235 | 0.002379 |
| PGK1 | 2.51E-07 | 0.609942 | 0.005059 |
| S100A2 | 3.03E-07 | 0.421746 | 0.006097 |
| GBE1 | 3.05E-07 | 0.318568 | 0.006149 |
| SEC61G | 4.59E-07 | 0.258519 | 0.009259 |
| PFKP | 5.71E-07 | 0.331277 | 0.011517 |
| FAM162A | 5.76E-07 | 0.682216 | 0.011619 |
| BEND5 | 6.43E-07 | 0.251597 | 0.012955 |
| LGALS3 | 1.98E-06 | 0.342243 | 0.997 |
