# Supplementary material for: Osteosarcoma tumors maintain intra-tumoral transcriptional heterogeneity during bone and lung colonization
Source: BMC Biol. 2023 Apr 27;21:98. doi: 10.1186/s12915-023-01593-3 (PMC10142502; doi:10.1186/s12915-023-01593-3)
Supplement: Supplementary file 2 — Additional file 2: Figure S1. Majority of the variance in our osteosarcoma datasets is dominated by cell cycle associated genes. [file 12915_2023_1593_MOESM2_ESM.pdf]

Figure S1

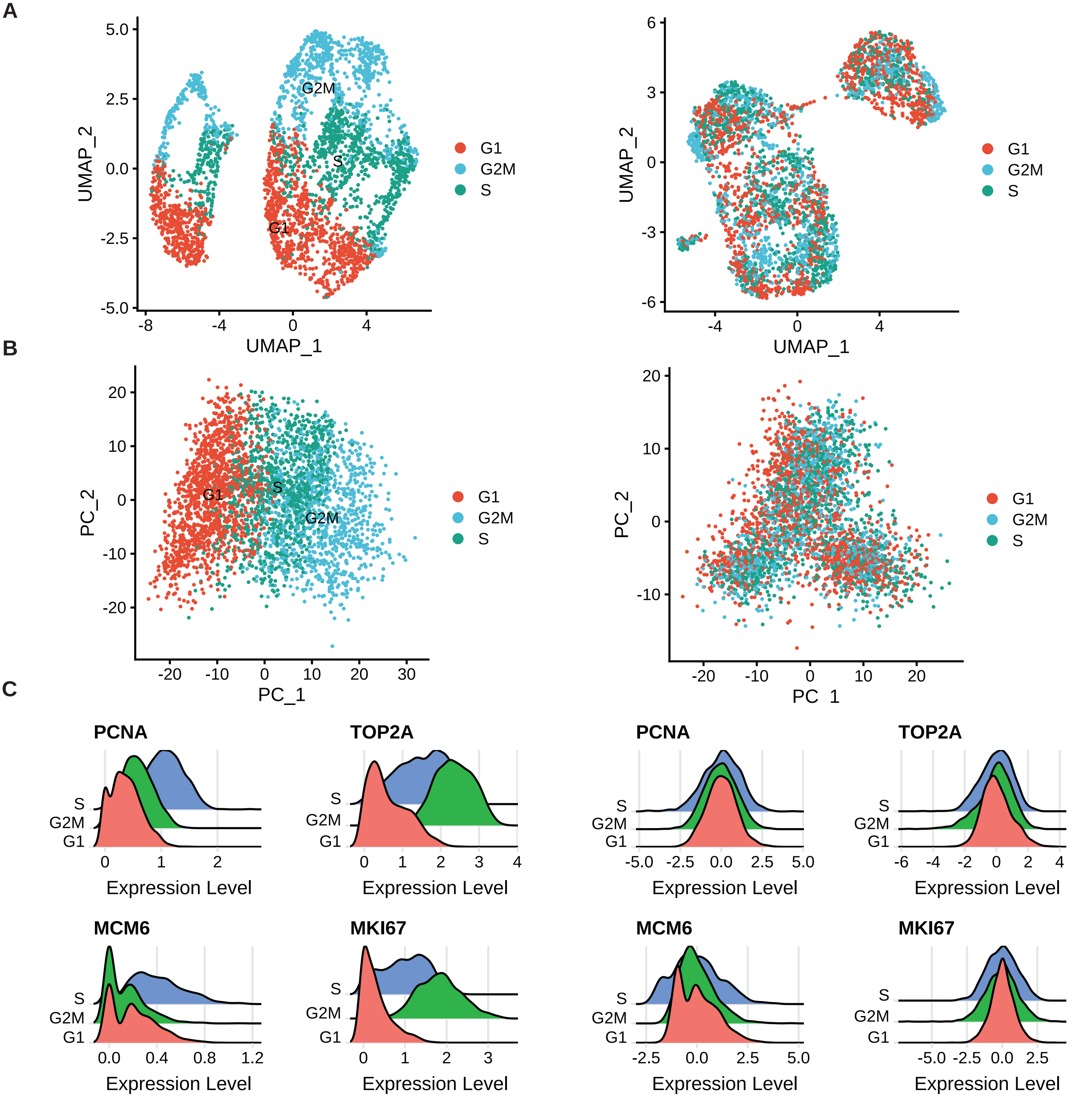

Figure S1. Majority of the variance in our osteosarcoma datasets is dominated by cell cycle associated genes. OS-17 cells were scored based on expression of G2/M and S phase markers, described in Tirosh et al. 2016[49]. A) UMAP analysis and B) PCA on cell cycle genes before cell cycle regression reveals that cells separate entirely by phase, confirmed using C) Ridge plots visualizing the distribution of cell cycle markers. After regressing out cell cycle scores, UMAP analysis and PCA on cell cycle genes show that cells no longer separate by phase. This is confirmed using Ridge plots of cell cycle markers. These plots are representative of all datasets.
