# Supplementary material for: Osteosarcoma tumors maintain intra-tumoral transcriptional heterogeneity during bone and lung colonization
Source: BMC Biol. 2023 Apr 27;21:98. doi: 10.1186/s12915-023-01593-3 (PMC10142502; doi:10.1186/s12915-023-01593-3)
Supplement: Supplementary file 3 — Additional file 3: Figure S2. In-depth analysis of clusters utilized in analyses. [file 12915_2023_1593_MOESM3_ESM.pdf]

Figure S2

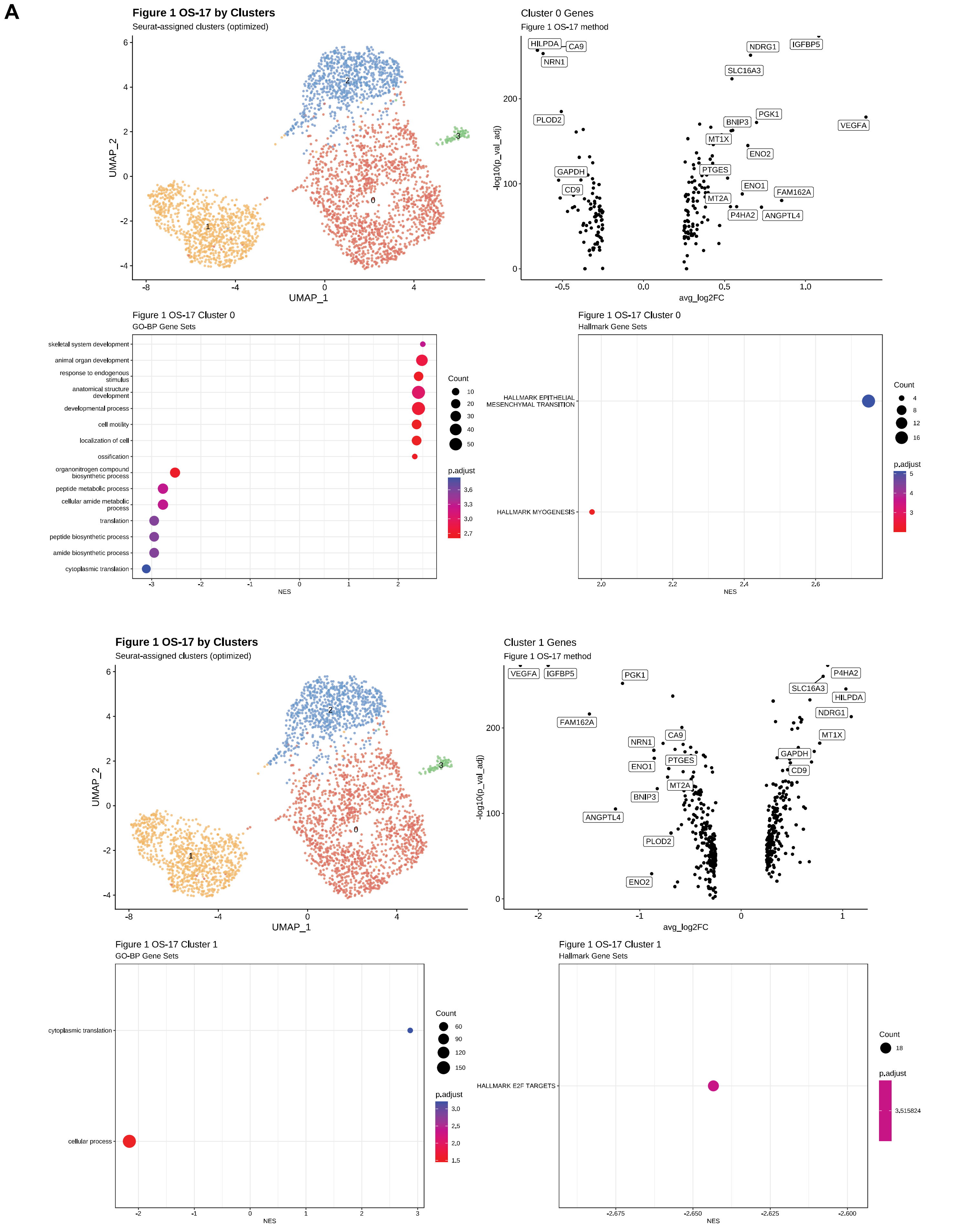

A
Figure 1 OS-17 by Clusters
Seurat-assigned clusters (optimized)
UMAP_2
UMAP_1
0
1
2
3
Cluster 0 Genes
Figure 1 OS-17 method
-log10(p_val_adj)
avg_log2FC
HILPDA
CA9
NRN1
NDRG1
IGFBP5
SLC16A3
PLOD2
PGK1
BNIP3
VEGFA
MT1X
ENO2
GAPDH
PTGES
CD9
ENO1
FAM162A
MT2A
P4HA2
ANGPTL4
Figure 1 OS-17 Cluster 0
GO-BP Gene Sets
skeletal system development
animal organ development
response to endogenous stimulus
anatomical structure development
developmental process
cell motility
localization of cell
ossification
organonitrogen compound biosynthetic process
peptide metabolic process
cellular amide metabolic process
translation
peptide biosynthetic process
amide biosynthetic process
cytoplasmic translation
NES
Count
p.adjust
Figure 1 OS-17 Cluster 0
Hallmark Gene Sets
HALLMARK EPITHELIAL MESENCHYMAL TRANSITION
HALLMARK MYOGENESIS
NES
Count
p.adjust
Figure 1 OS-17 by Clusters
Seurat-assigned clusters (optimized)
UMAP_2
UMAP_1
Cluster 1 Genes
Figure 1 OS-17 method
-log10(p_val_adj)
avg_log2FC
VEGFA
IGFBP5
PGK1
P4HA2
SLC16A3
HILPDA
NDRG1
FAM162A
CA9
MT1X
NRN1
GAPDH
PTGES
ENO1
CD9
MT2A
BNIP3
ANGPTL4
PLOD2
ENO2
Figure 1 OS-17 Cluster 1
GO-BP Gene Sets
cytoplasmic translation
cellular process
NES
Count
p.adjust
Figure 1 OS-17 Cluster 1
Hallmark Gene Sets
HALLMARK E2F TARGETS
NES
Count
p.adjust
3,515824

Figure S2 continued

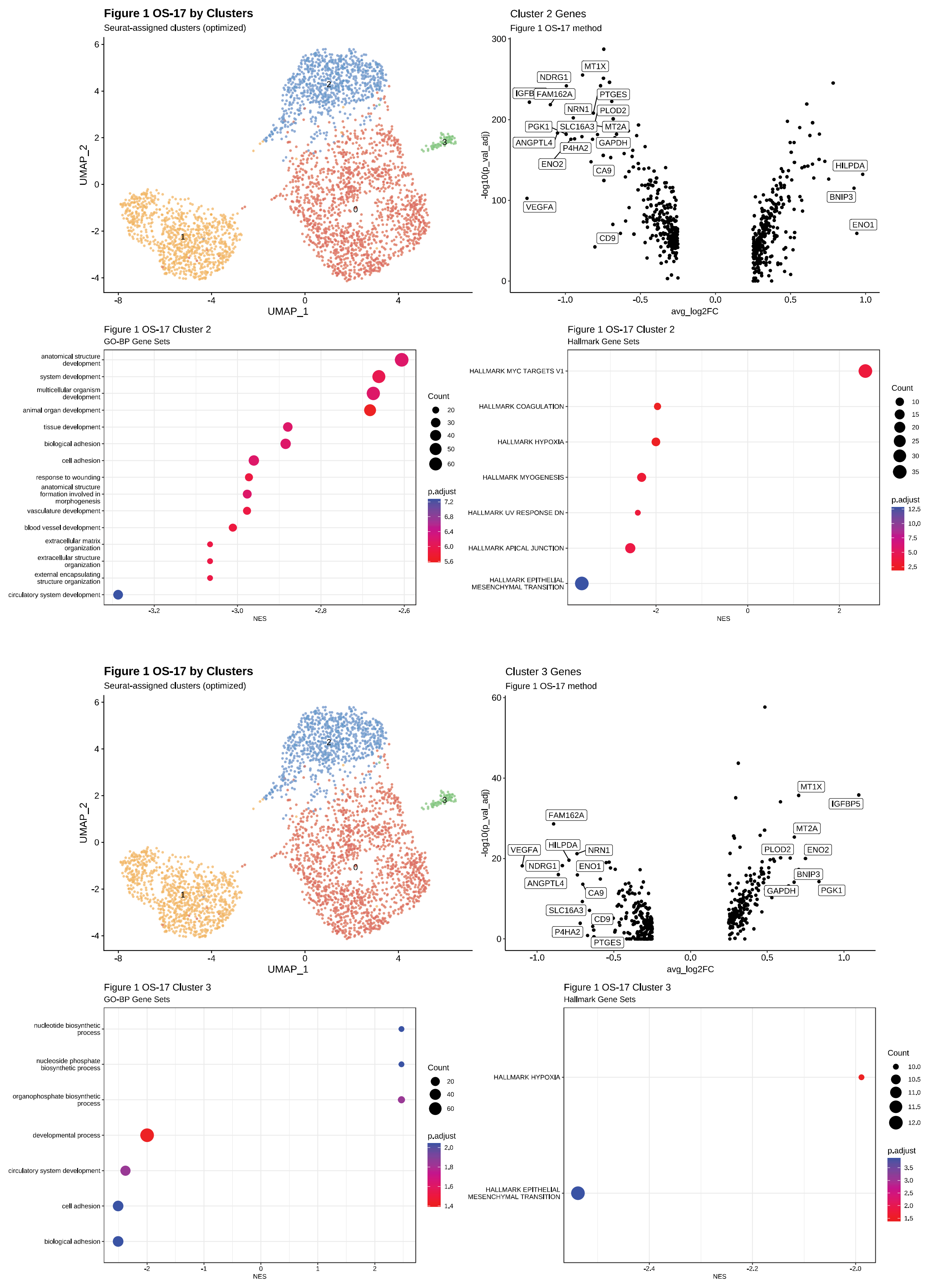

Figure S2 continued

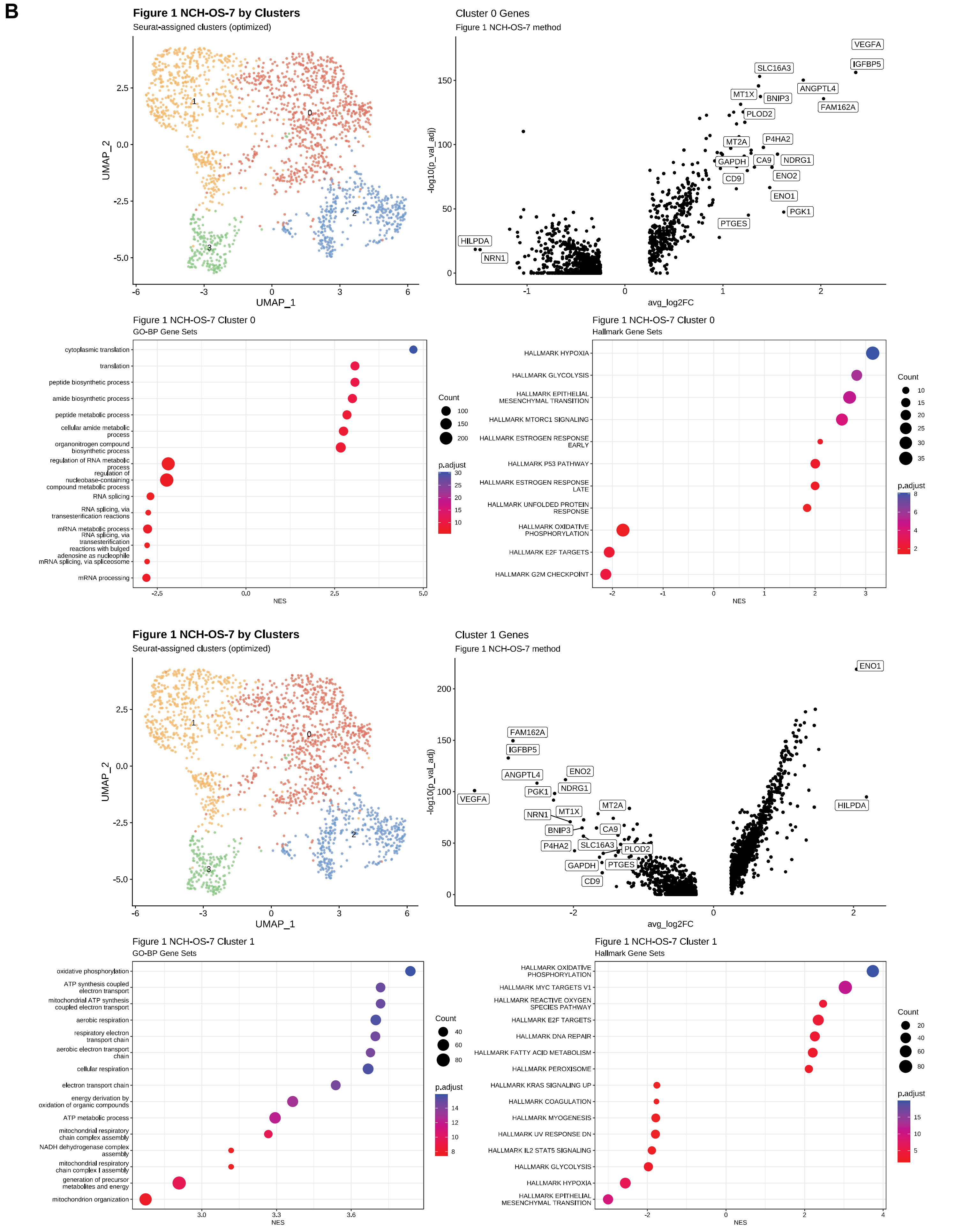

Figure S2 continued

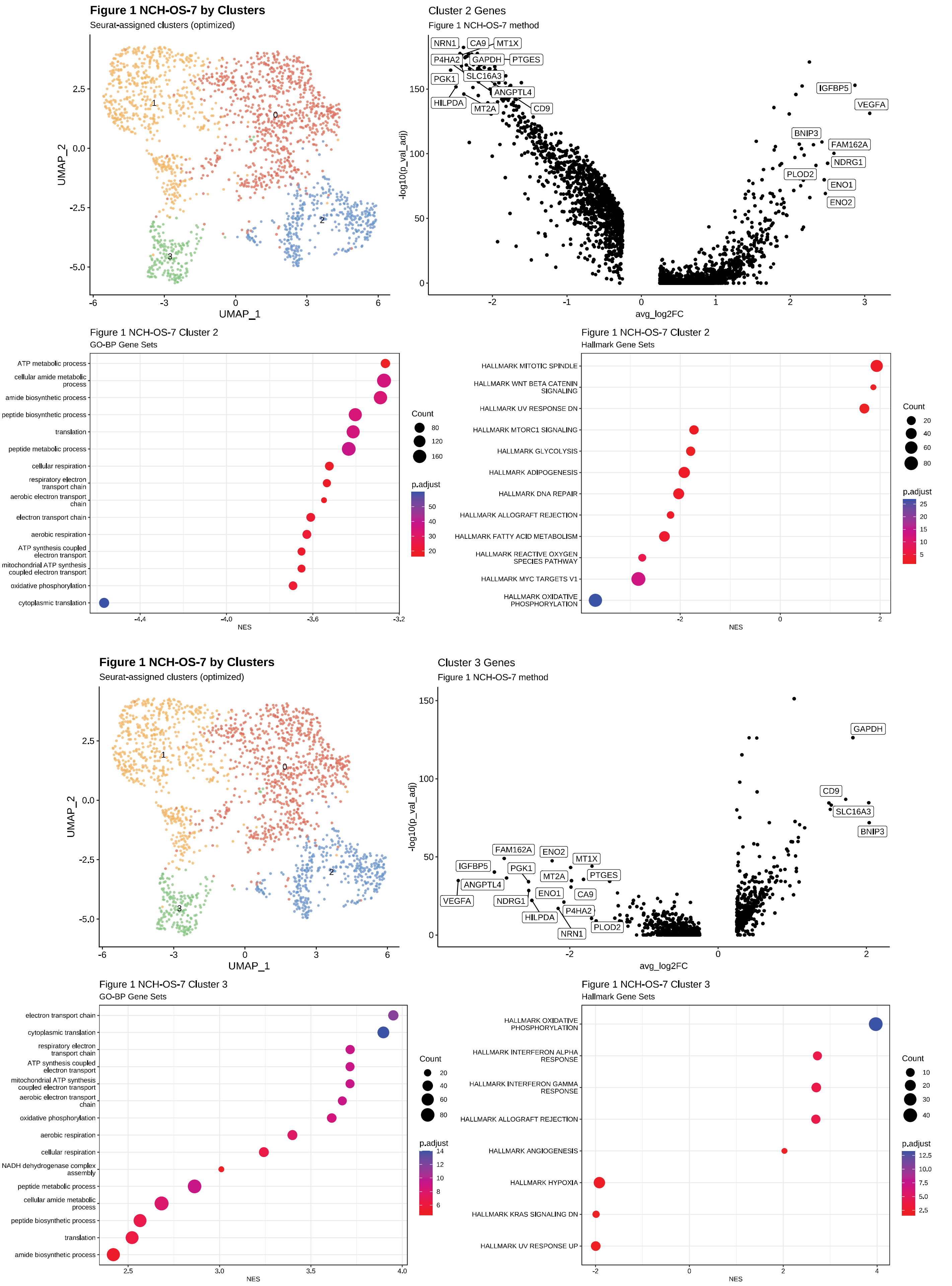

Figure S2 continued

C

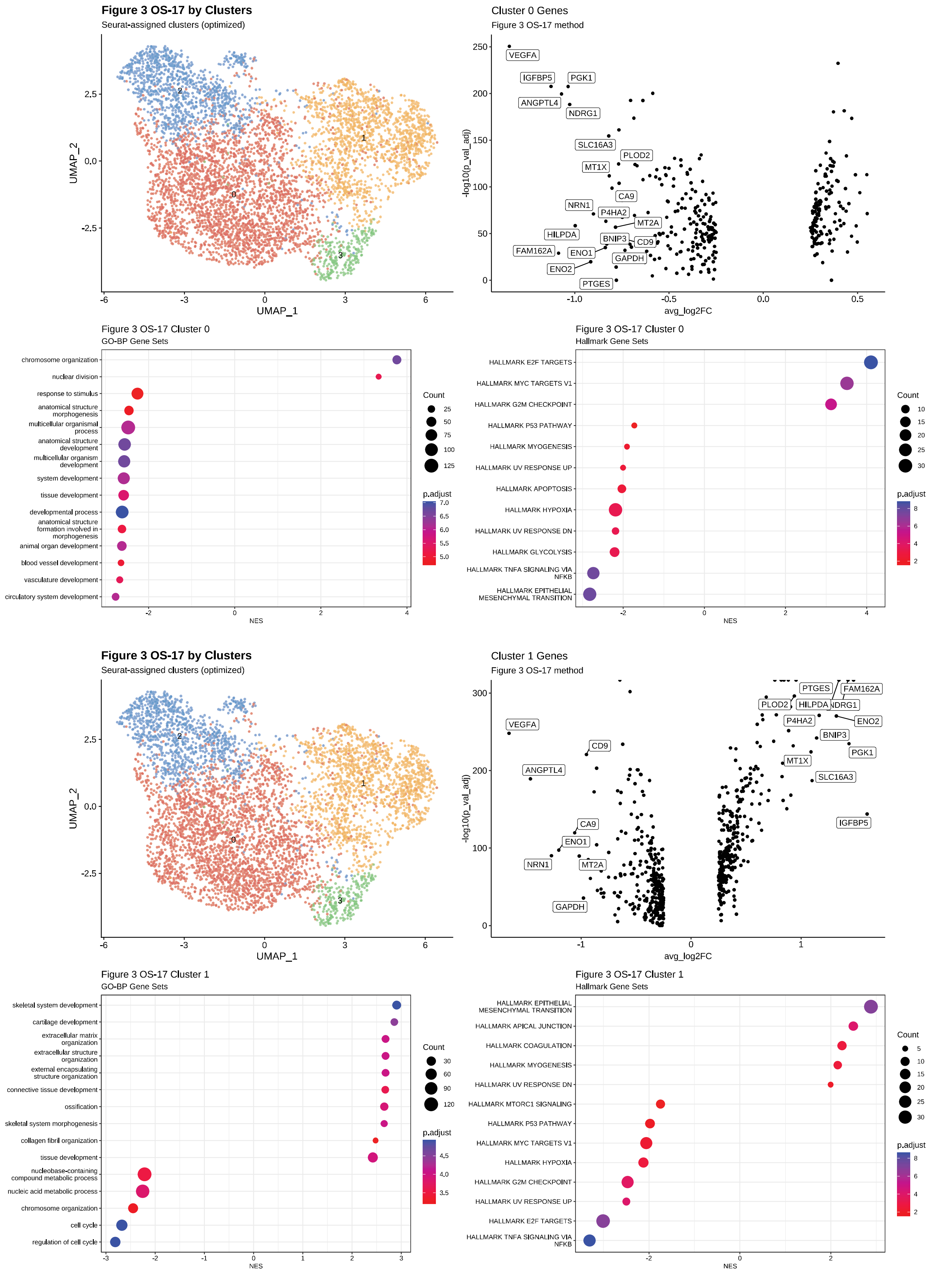

Figure S2 continued

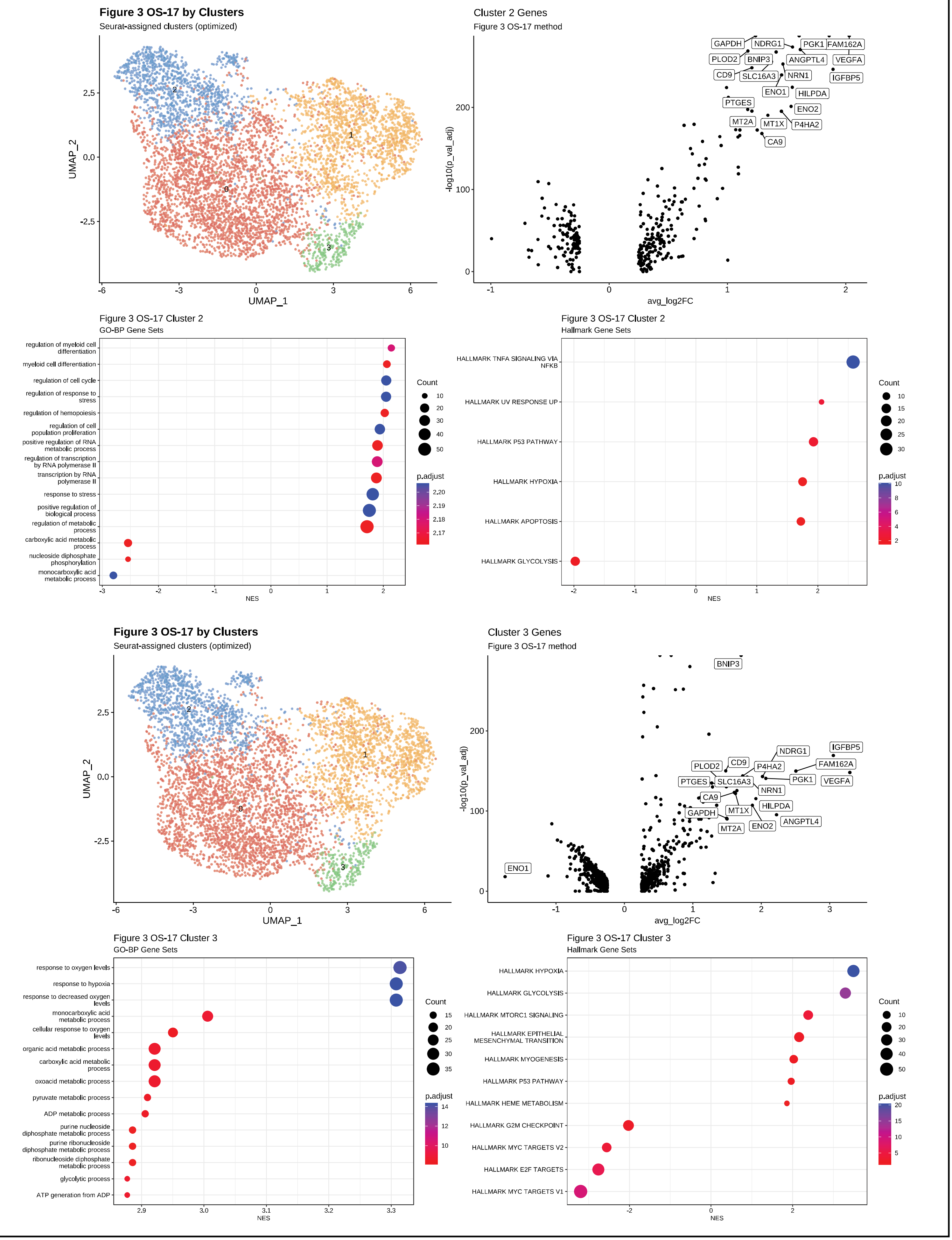

Figure S2 continued

D

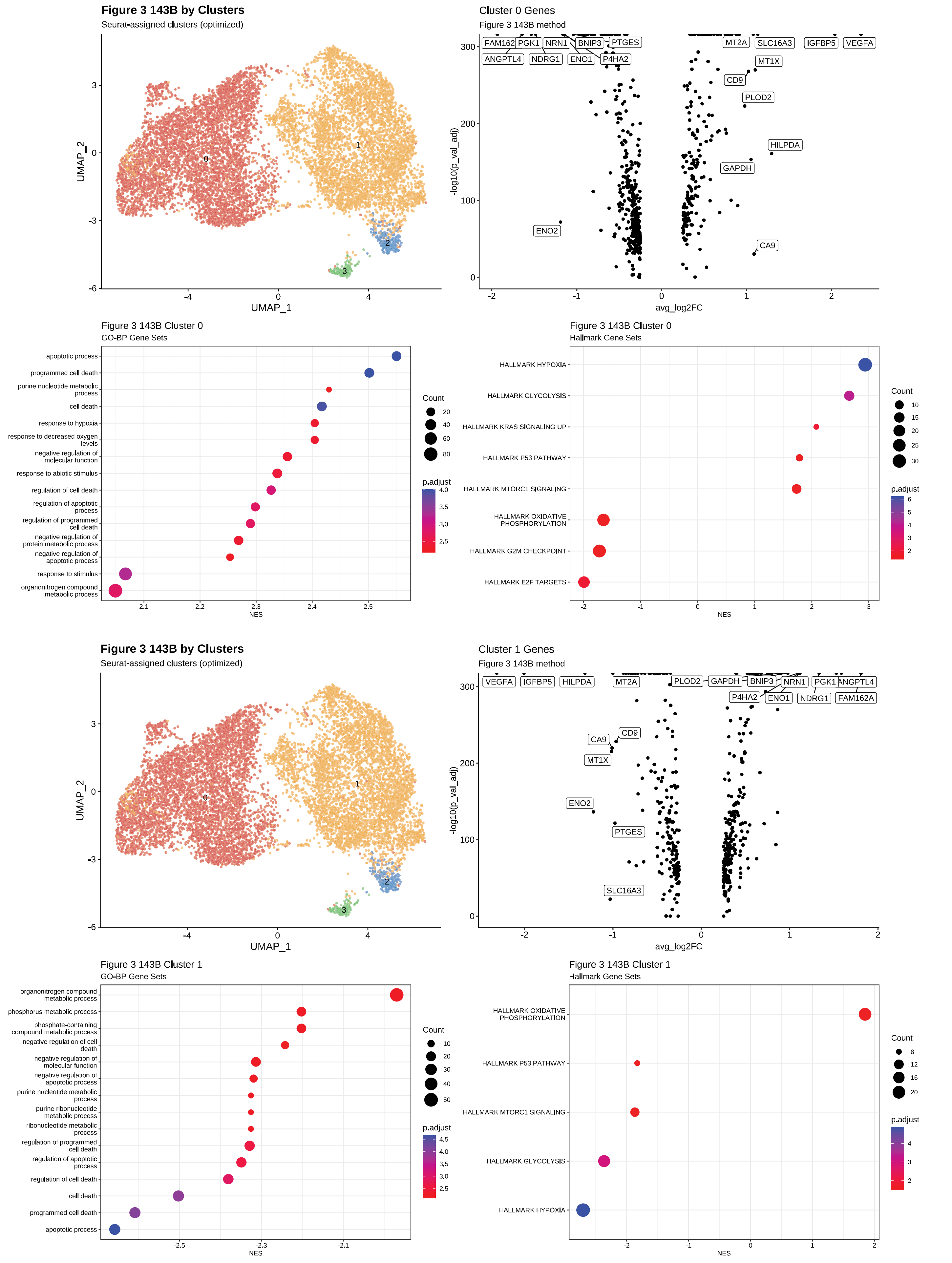

Figure S2 continued

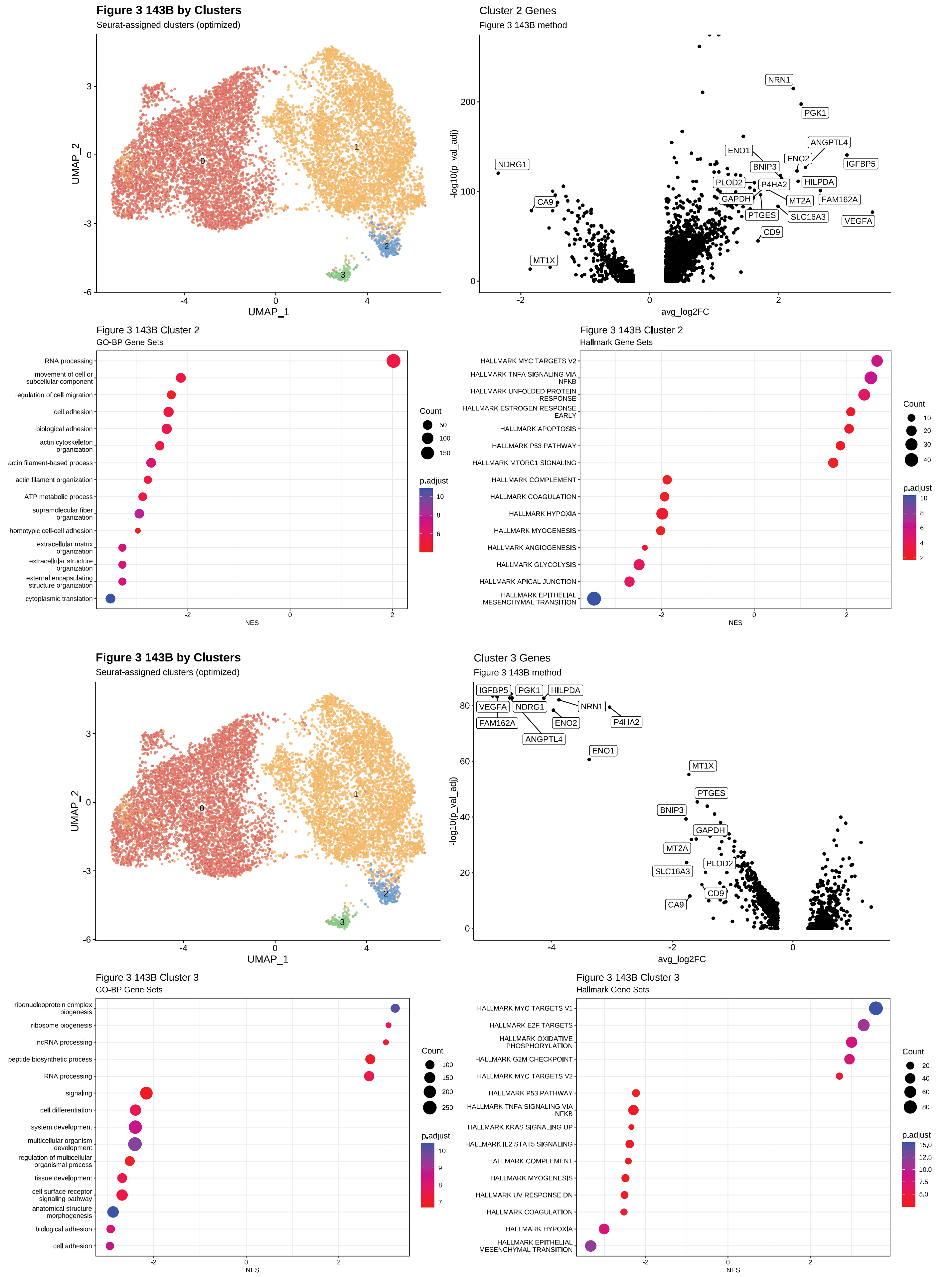

Figure S2 continued

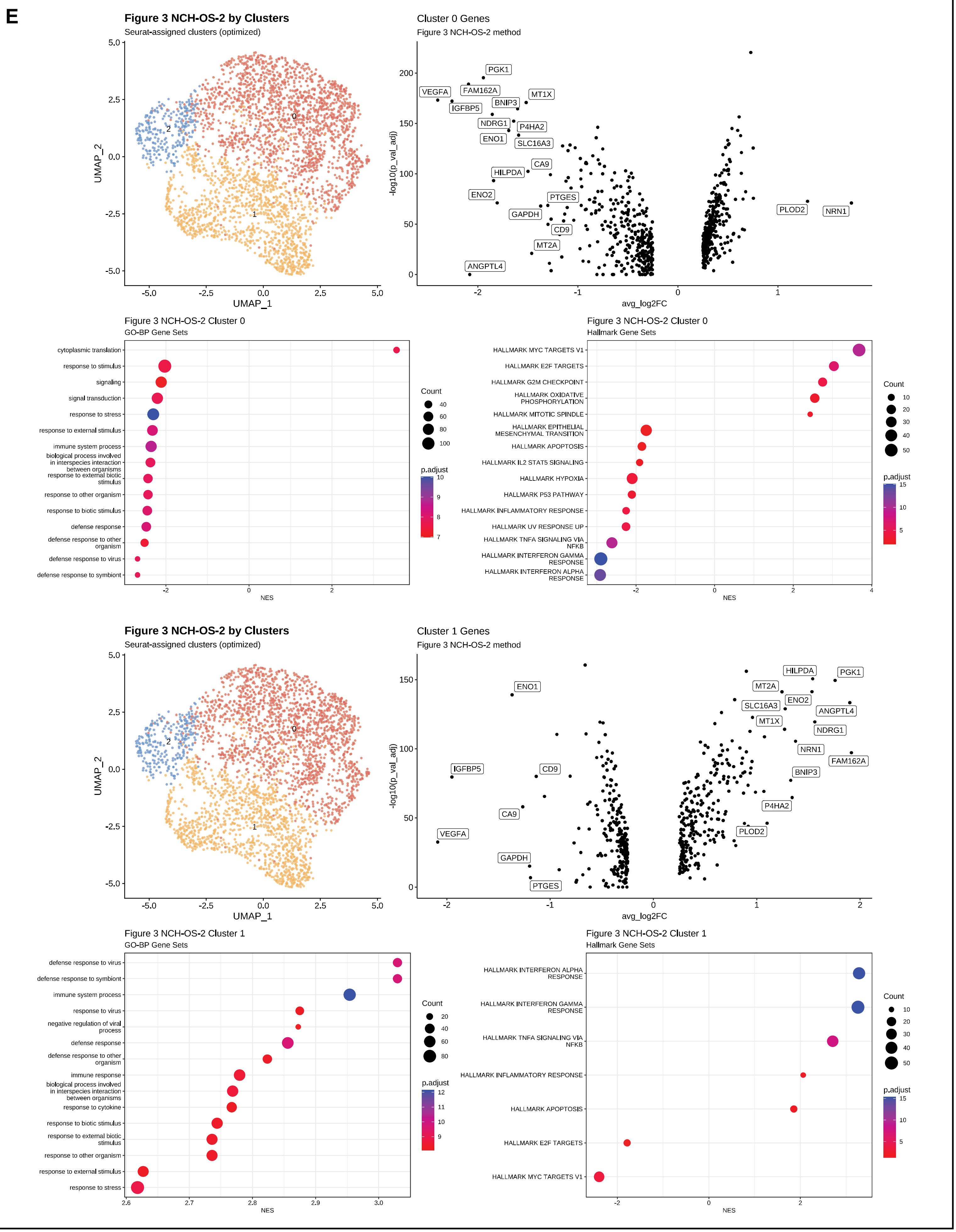

Figure S2 continued

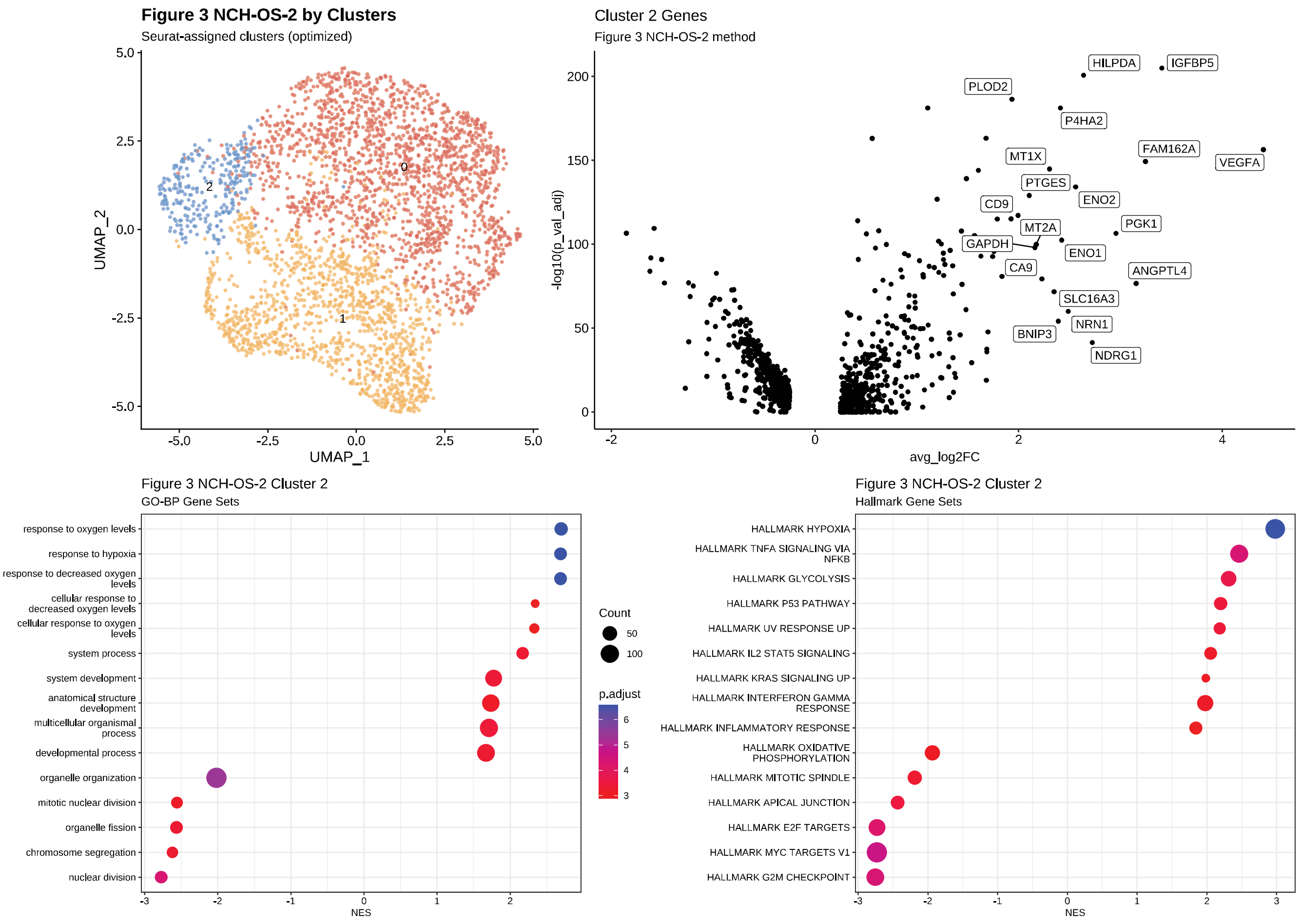

F

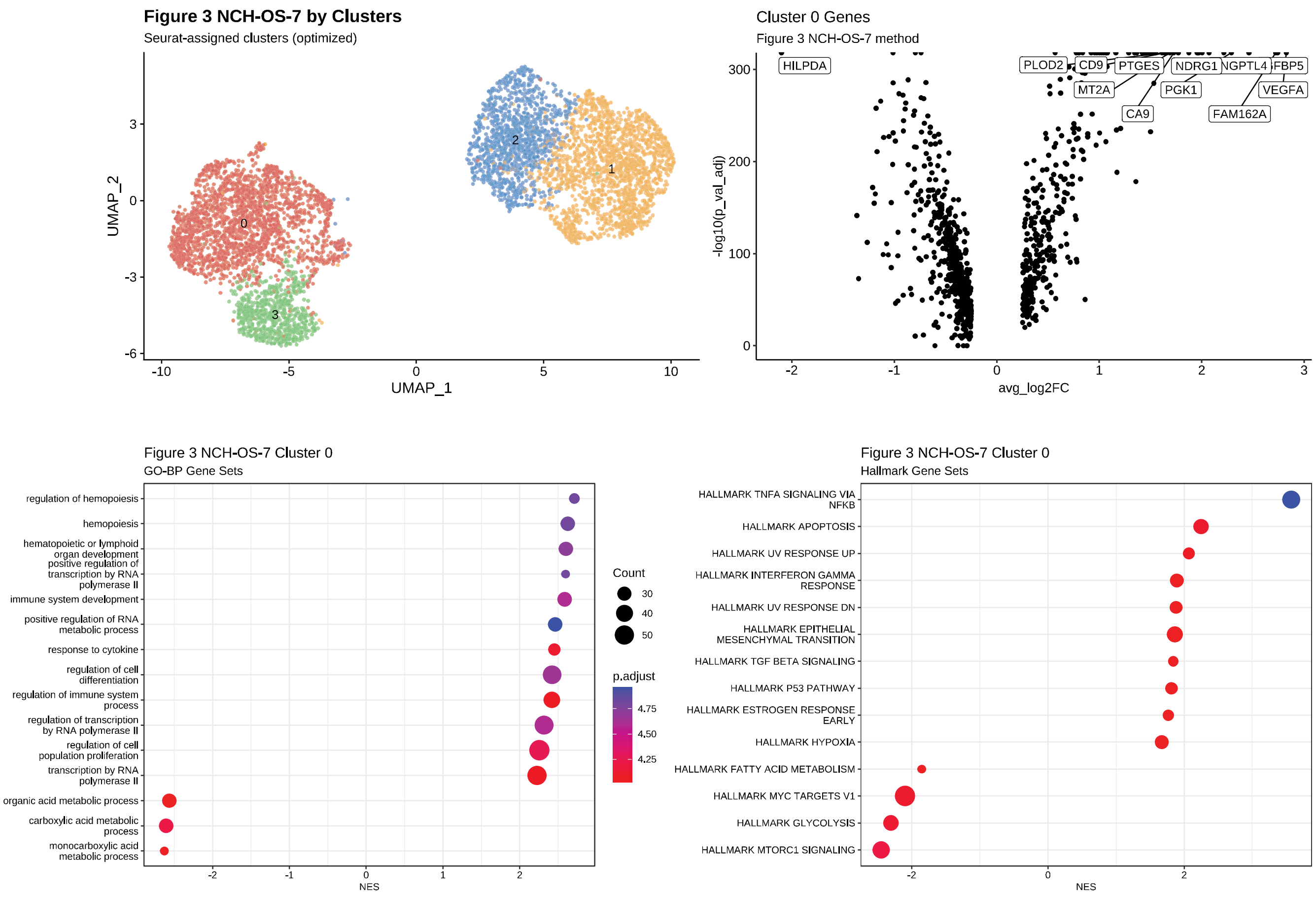

Figure S2 continued

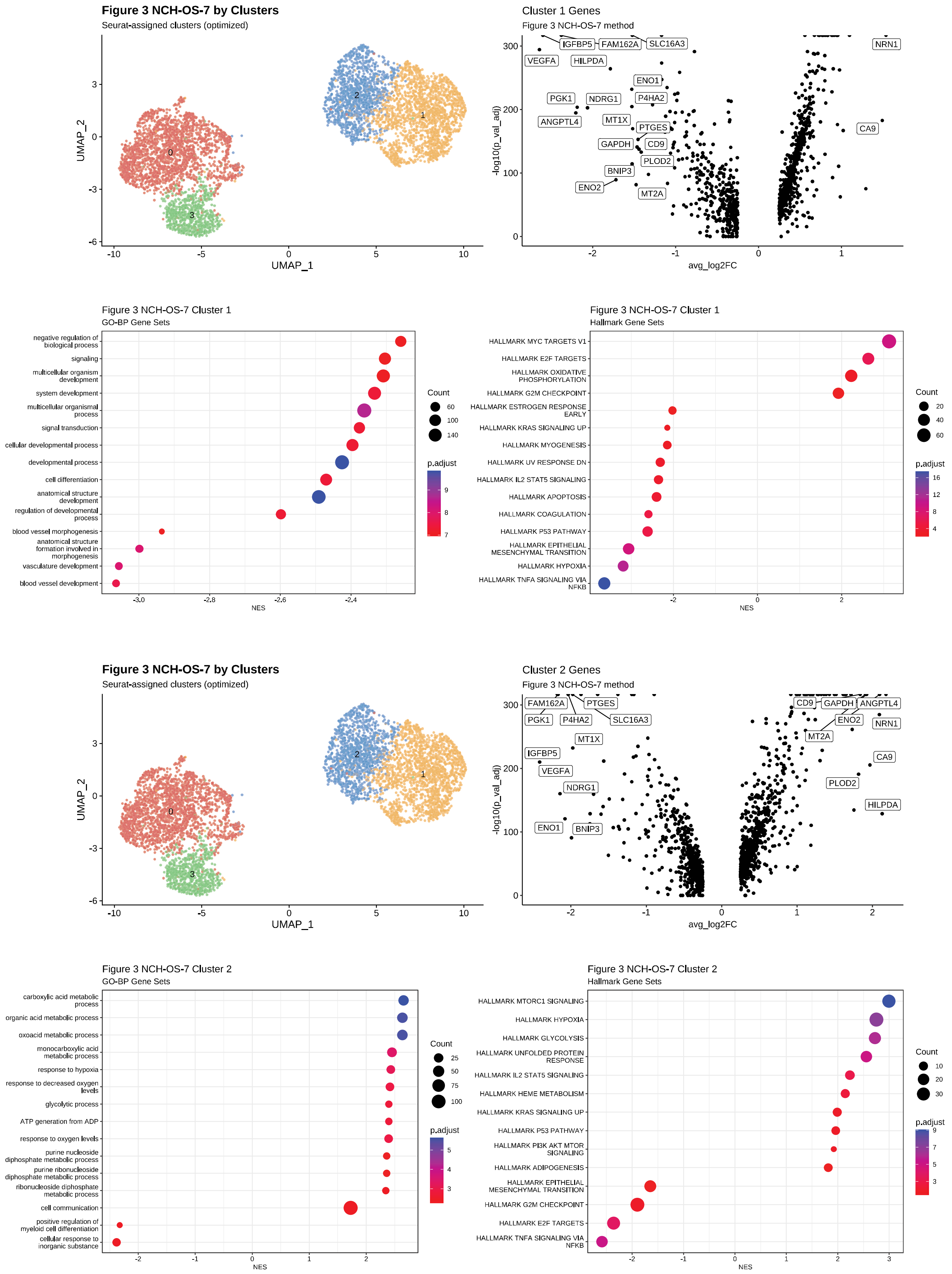

Figure S2 continued

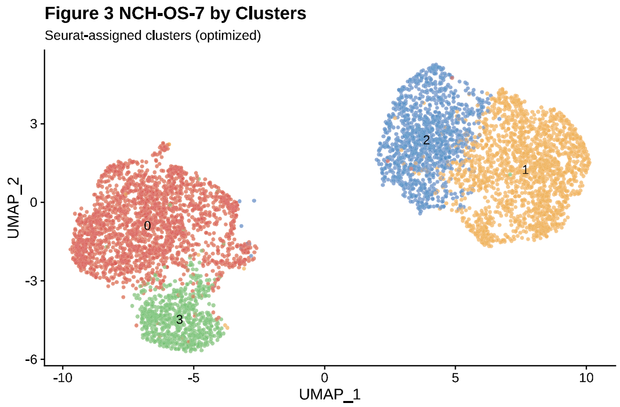

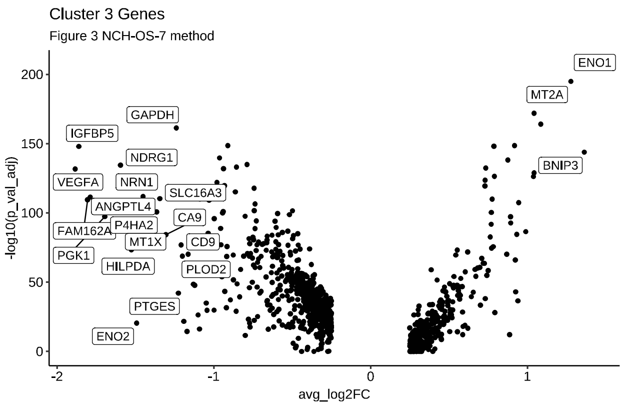

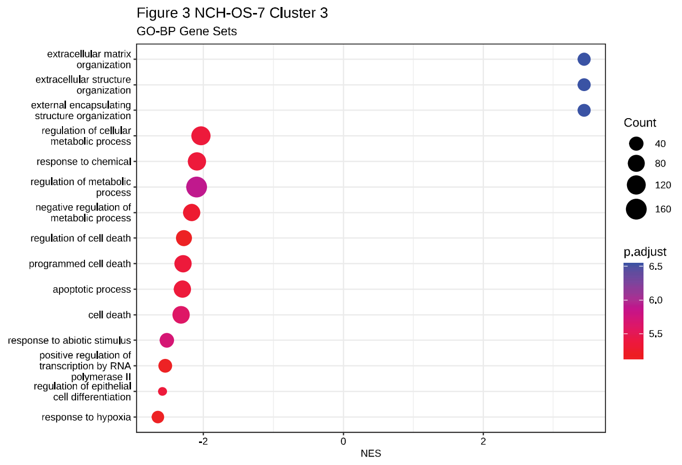

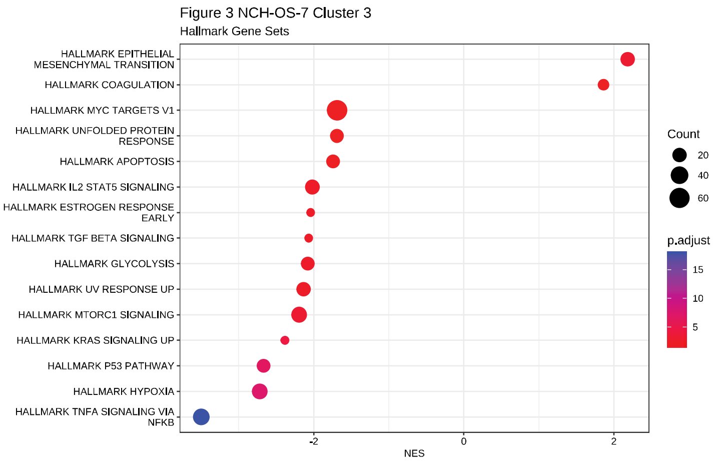

G

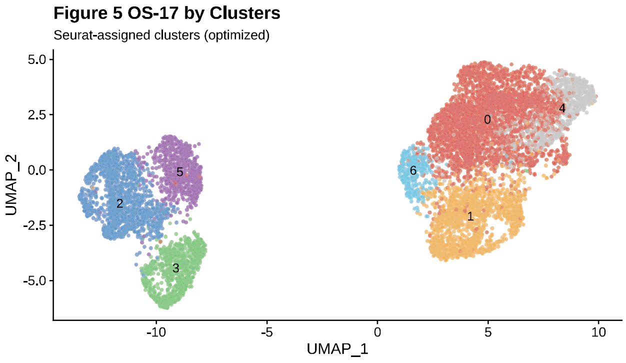

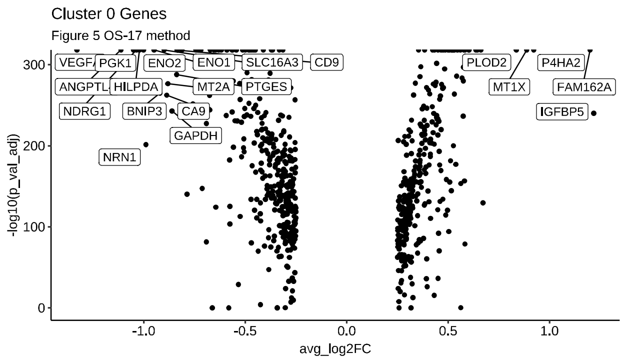

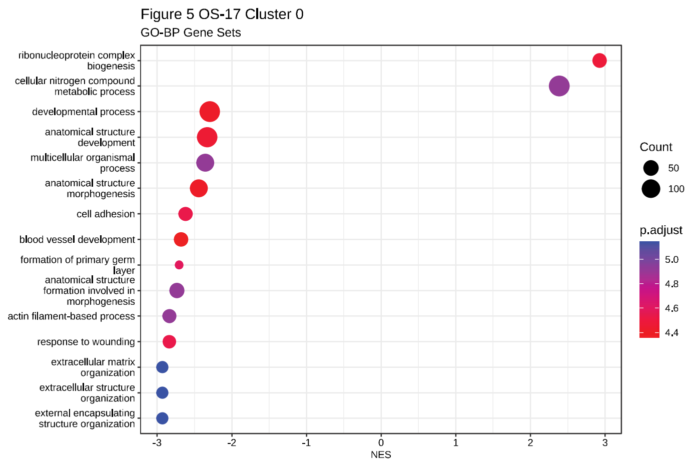

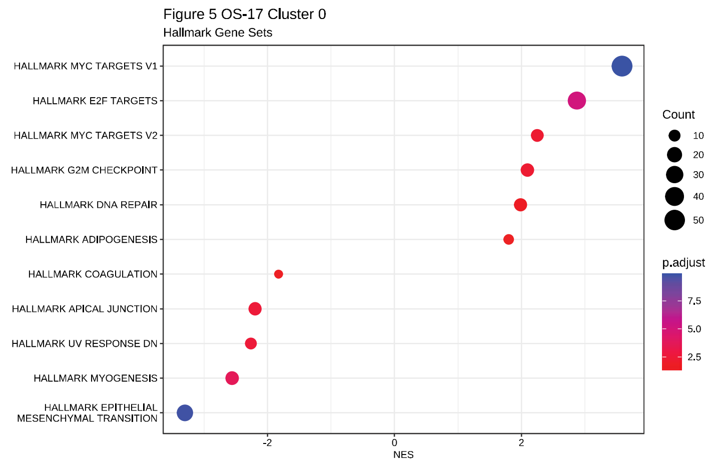

Figure S2 continued

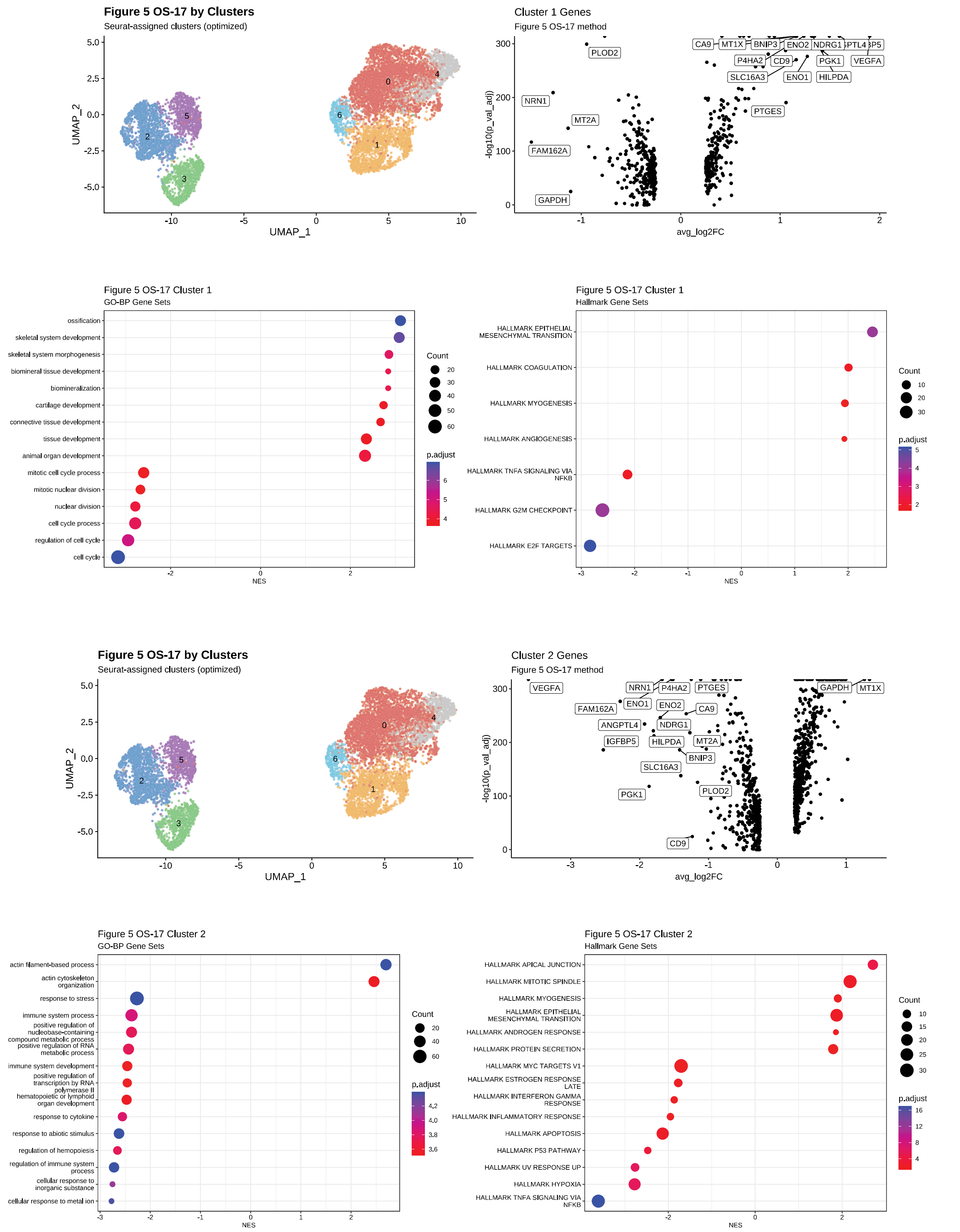

Figure S2 continued

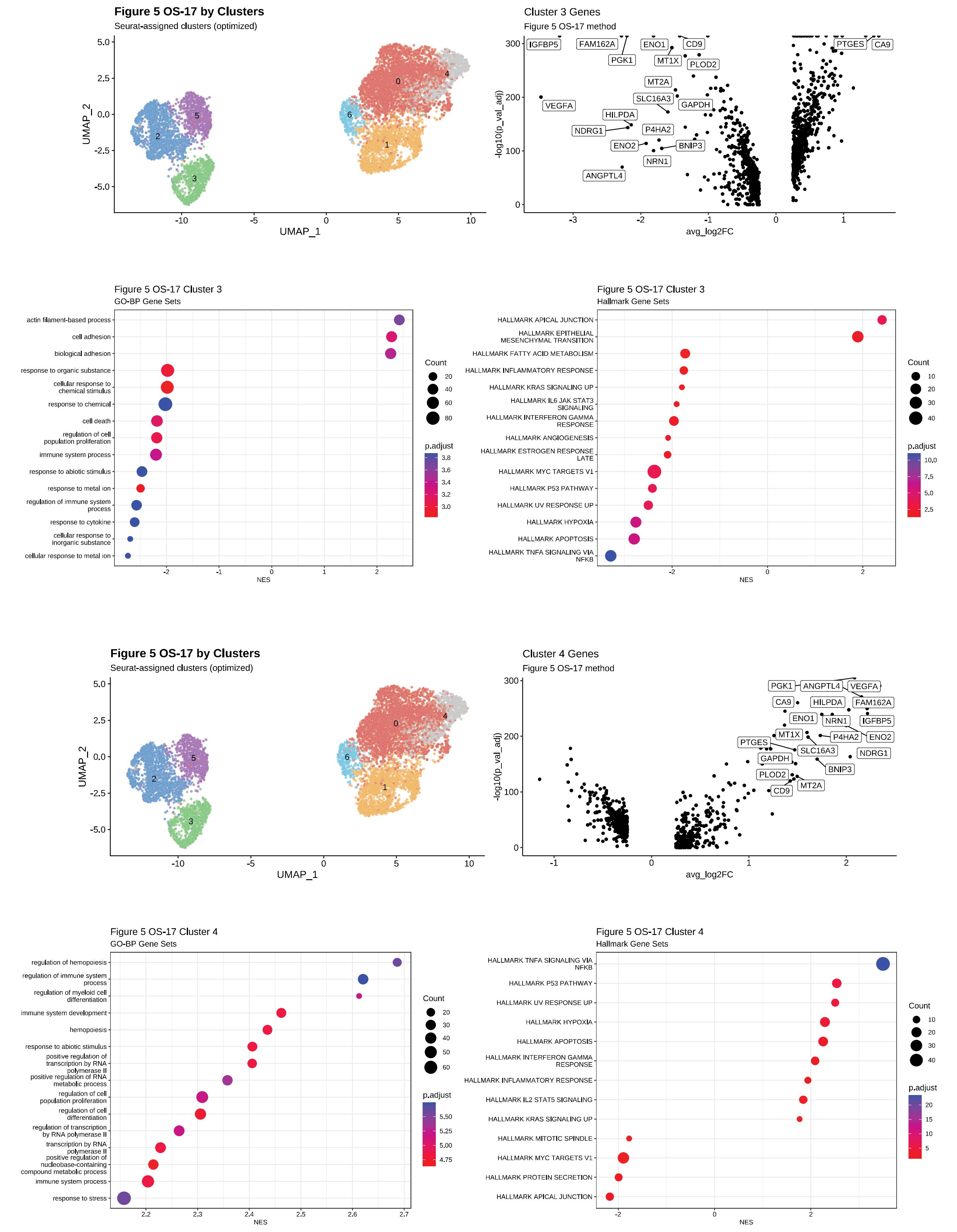

Figure S2 continued

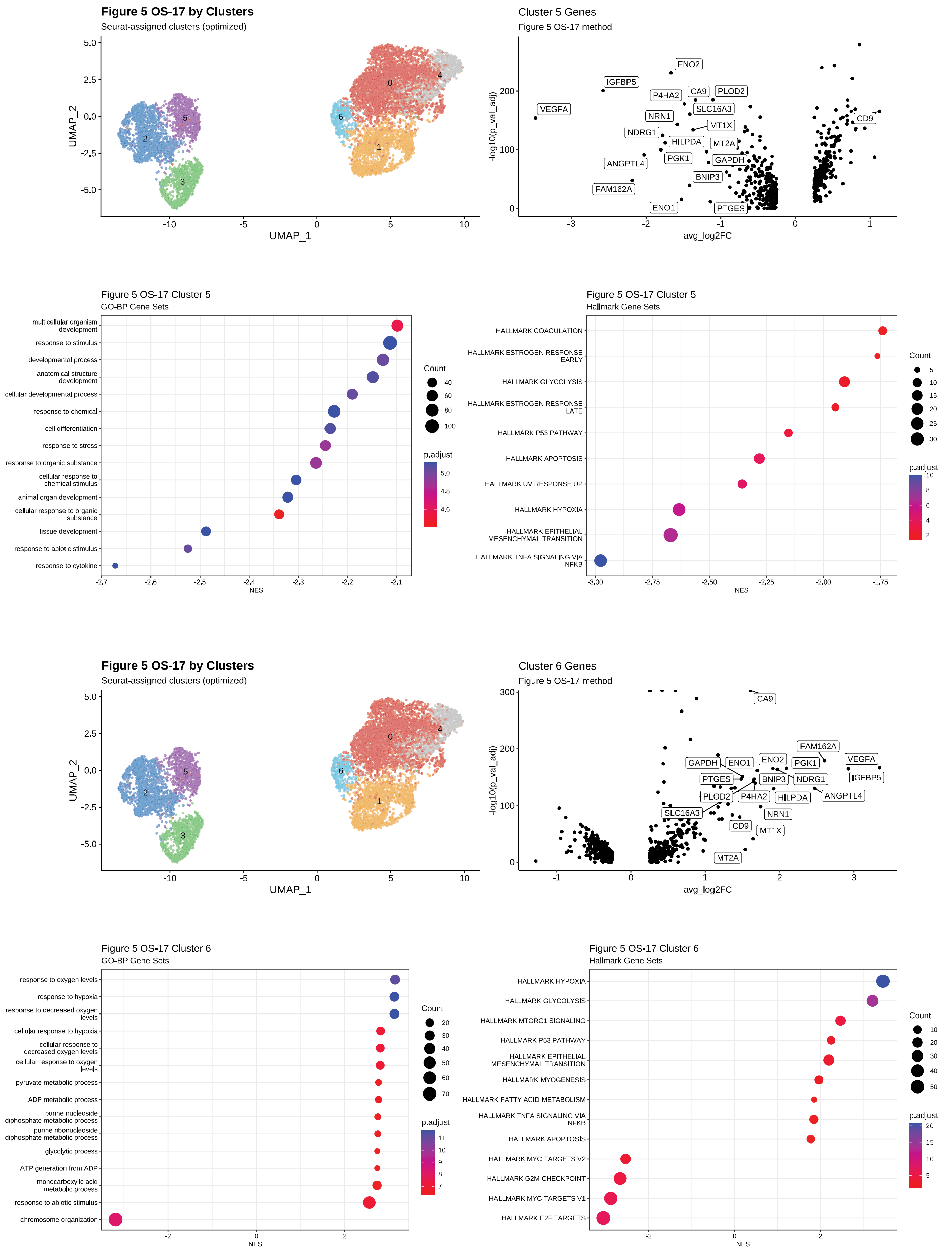

Figure S2 continued

Figure S2. In-depth analysis of clusters utilized in analyses. A) OS-17 cell culture and B) NCH-OS-2 flank cluster analysis (Figure 1). C-F) Merged tibia and lung cluster analysis for OS-17, 143B, NCH-OS-2, and NCH-OS-7, respectively (Figure 3). G) Lineage-tagged OS-17 cell culture, tibia, and lung cluster analysis (Figure 5). The upper left of each analysis displays all clusters for the dataset. The upper right shows a volcano plot analysis, where the top differentially expressed genes for the cluster of interest, compared to all others in the dataset, are shown. DotPlots on the bottom left depict the significantly upregulated GO BioProcesses and GO MolFunction gene lists in the cluster of interest from the differentially expressed gene list for the cluster. Bar plot on the bottom left shows upregulated msigdb Hallmark pathways from the differentially expressed gene list for the cluster. The Wilcoxon rank sum test, or “wilcox” statistical method, was used to generate differentially expressed gene lists.
