# Supplementary material for: Osteosarcoma tumors maintain intra-tumoral transcriptional heterogeneity during bone and lung colonization
Source: BMC Biol. 2023 Apr 27;21:98. doi: 10.1186/s12915-023-01593-3 (PMC10142502; doi:10.1186/s12915-023-01593-3)
Supplement: Supplementary file 4 — Additional file 4: Figure S3. Downregulated pathways across all cell line- and PDX-derived tumors. [file 12915_2023_1593_MOESM4_ESM.pdf]

Figure S3

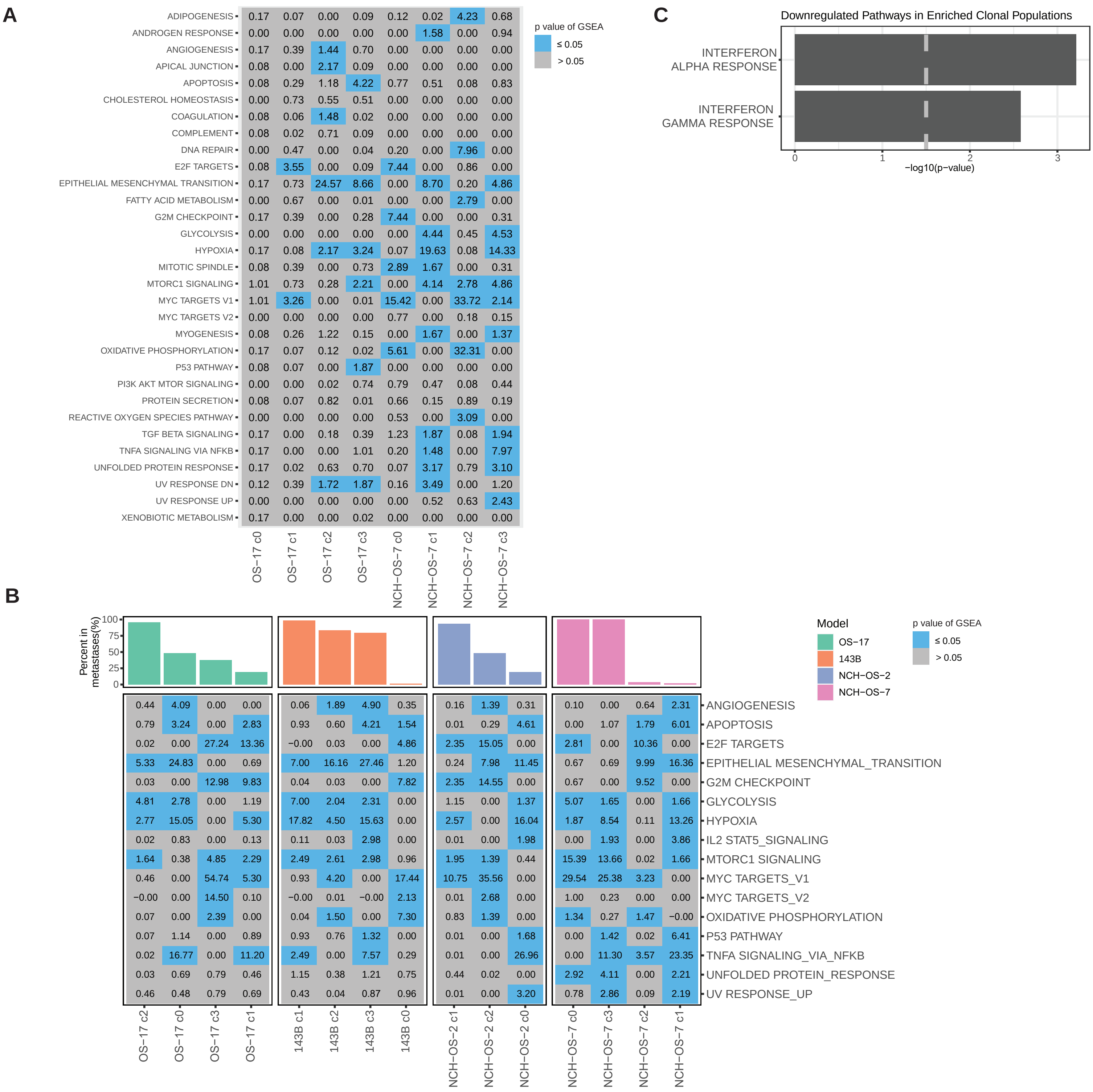

Figure S3. Downregulated pathways across all cell line- and PDX-derived tumors. A) Pathway enrichment analysis for hallmark gene sets associated with genes downregulated in distinct clusters identified in OS-17 and NCH-OS-7 osteosarcoma models (Figure 1C). B) Heterogeneity in downregulated hallmark pathway gene sets. Bar plots show percentage of cells identified per cluster in lung lesions. C) Pathway enrichment analysis for hallmark gene sets comparing enriched clones relative to the remaining lung-colonizing tumor cells identifies genes related to interferon alpha response and interferon gamma response to be significantly downregulated. For A-B, we used a pathway enrichment analysis for hallmark gene sets using genes differentially downregulated in each cluster relative to every other cluster within the same model. P values were adjusted for multiple comparisons. Boxes in grey identify non-significant pathway enrichments, whereas boxes in blue identify statistically significant enrichments. For C, only statistically significant (p<0.05) downregulated pathways in enriched clones relative to the remaining lung-colonizing tumor cells are shown.
