# Supplementary material for: Osteosarcoma tumors maintain intra-tumoral transcriptional heterogeneity during bone and lung colonization
Source: BMC Biol. 2023 Apr 27;21:98. doi: 10.1186/s12915-023-01593-3 (PMC10142502; doi:10.1186/s12915-023-01593-3)
Supplement: Supplementary file 5 — Additional file 5: Figure S4. Mouse osteosarcoma models share distinct transcriptional profiles with human osteosarcoma models during tibia and lung colonization. [file 12915_2023_1593_MOESM5_ESM.pdf]

Figure S4

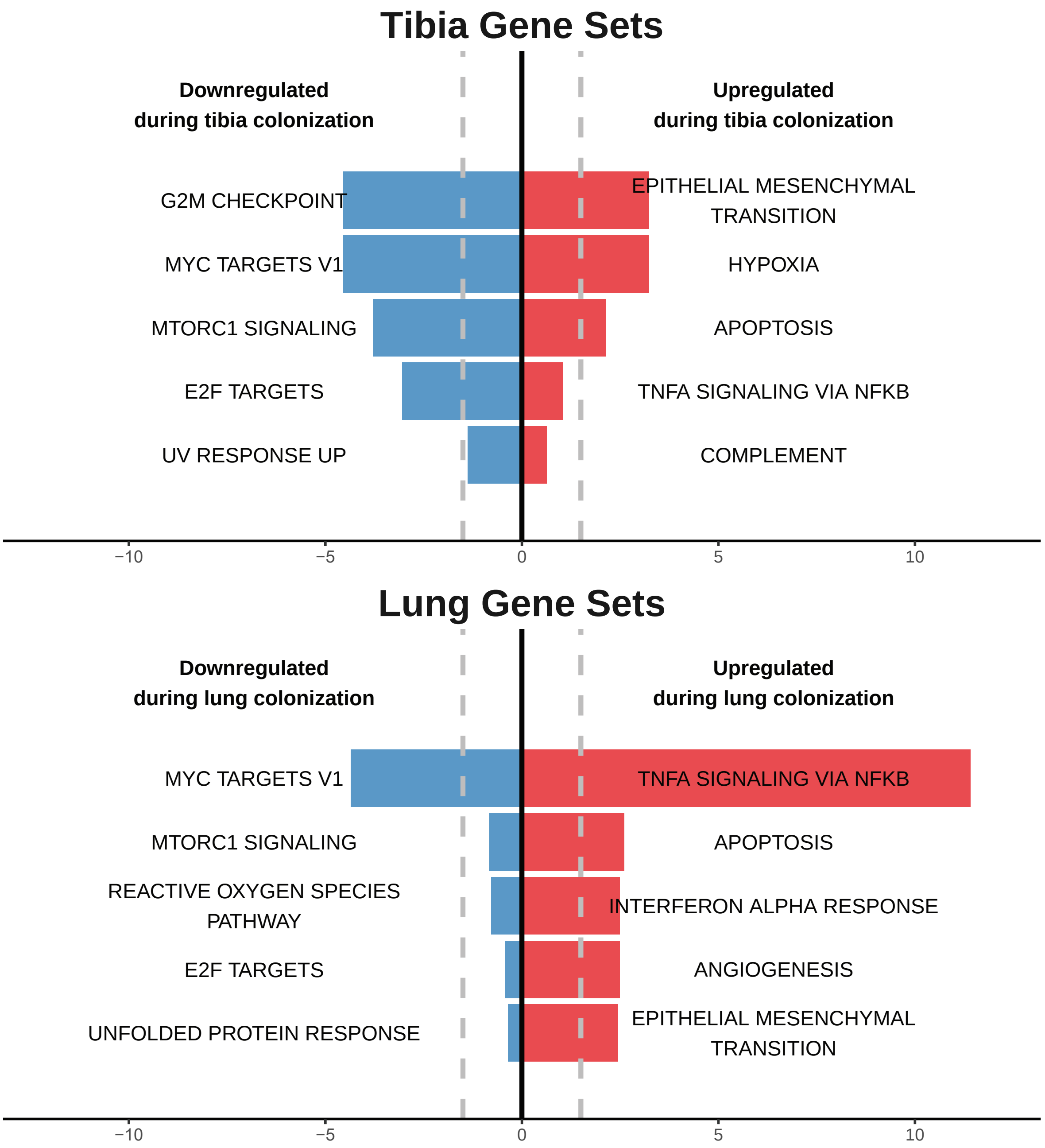

Figure S4. Mouse osteosarcoma models share distinct transcriptional profiles with human osteosarcoma models during tibia and lung colonization. K7M2 and F420 mouse models of osteosarcoma were added to the pathway enrichment analysis with the human models (OS-17, 143B, NCH-OS-2, NCH-OS-7) to determine conserved enriched pathways between mouse and human models. Pathway enrichment analysis with adjusted p values for hallmark gene sets associated with shared genes from the overlap of differentially expressed genes that are up- or down-regulated in tibia or lung lesions relative to corresponding starting population of cells (cell line or PDX flank tumors).
