# Supplementary material for: Osteosarcoma tumors maintain intra-tumoral transcriptional heterogeneity during bone and lung colonization
Source: BMC Biol. 2023 Apr 27;21:98. doi: 10.1186/s12915-023-01593-3 (PMC10142502; doi:10.1186/s12915-023-01593-3)
Supplement: Supplementary file 6 — Additional file 6: Figure S5. Osteoblast cells demonstrate phenotypic heterogeneity. [file 12915_2023_1593_MOESM6_ESM.pdf]

Figure S5

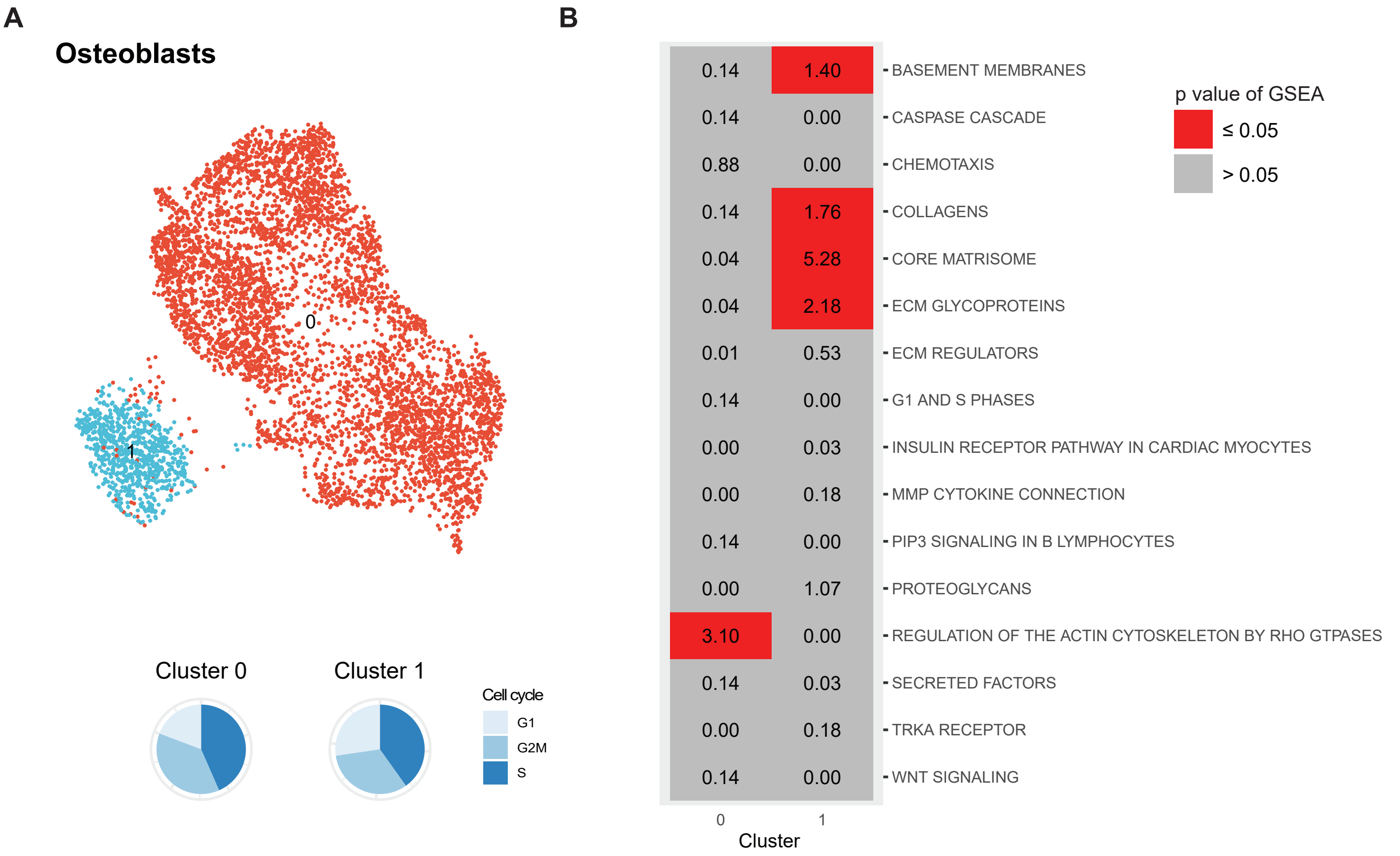

Figure S5. Osteoblast cells demonstrate phenotypic heterogeneity. A) UMAP analysis of normal human osteoblast cells in cell culture. B) Pathway enrichment analysis against msigdb C2 canonical pathways on genes differentially upregulated in each cluster relative remaining cells in the dataset.
