# Supplementary material for: Osteosarcoma tumors maintain intra-tumoral transcriptional heterogeneity during bone and lung colonization
Source: BMC Biol. 2023 Apr 27;21:98. doi: 10.1186/s12915-023-01593-3 (PMC10142502; doi:10.1186/s12915-023-01593-3)
Supplement: Supplementary file 7 — Additional file 7: Figure S6. Additional mouse models of osteosarcoma retain phenotypic heterogeneity despite adaptive changes in response to changing microenvironments. [file 12915_2023_1593_MOESM7_ESM.pdf]

Figure S6

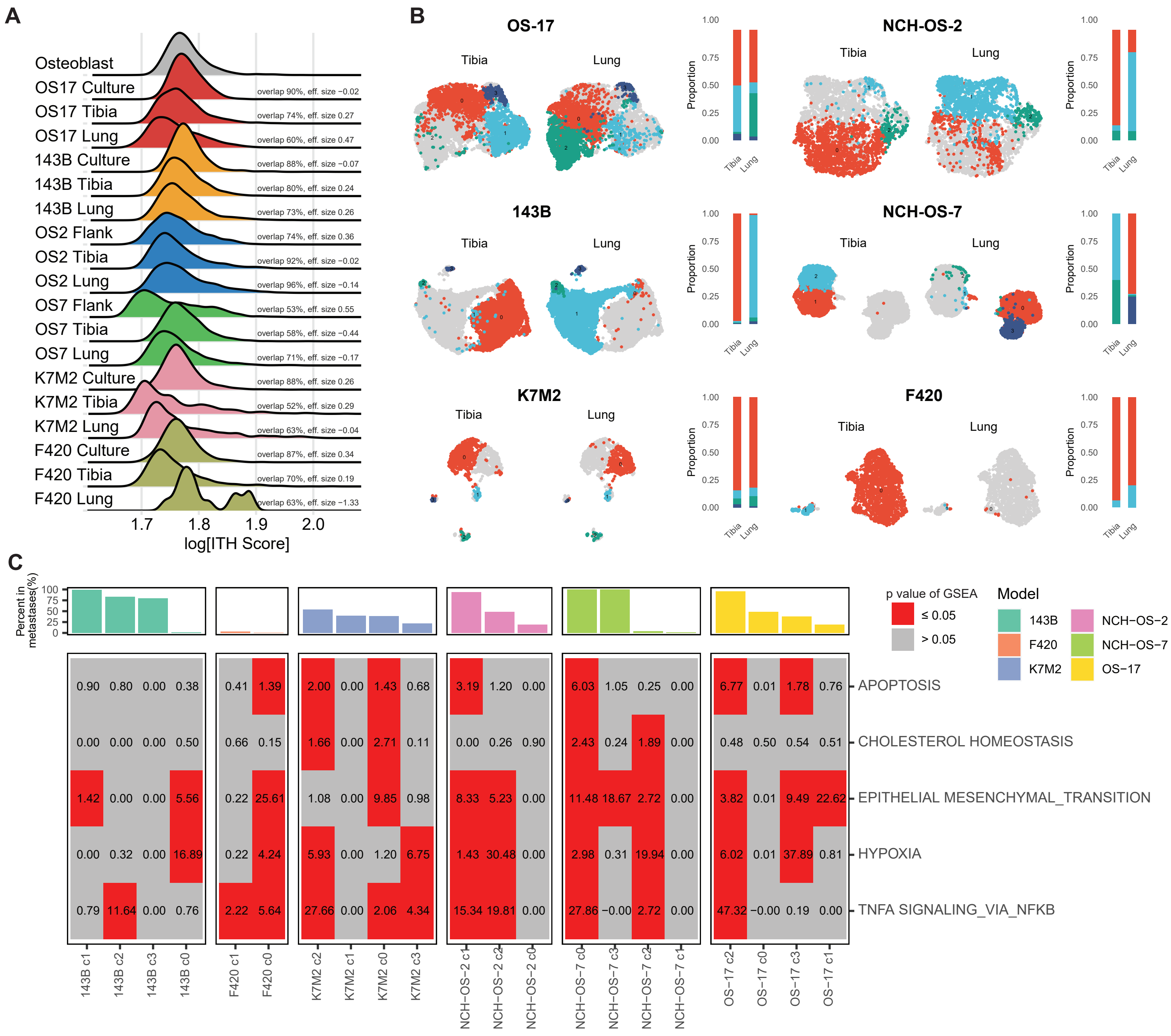

Figure S6. Additional mouse models of osteosarcoma retain phenotypic heterogeneity despite adaptive changes in response to changing microenvironments. A) Mouse osteosarcoma models maintained overall heterogeneity with a high degree of overlap between conditions. The ridge plot shows ITH scores, which represent the gene expression “distance” between each tumor cell within a sample and all of the other tumor cells from that same sample. The overlap statistic describes the total percentage of overlap in the observed distributions between two samples. B) UMAP analysis for merged tibia- and lung-colonizing tumor samples in each of the six models, including the mouse osteosarcoma models. Cells in grey represent remaining cells in merged sample. Cluster enrichment analysis shows distribution of cells in each cluster in the two microenvironment conditions (tibia, lung). While some cells in the tibia and lung lesions adopted shared phenotypes, others adopted distinct phenotypes. C) Glycolysis activation is not shared in mouse osteosarcoma models, but hypoxia heterogeneity is maintained. We used a pathway enrichment analysis for hallmark gene sets using genes differentially upregulated in each cluster relative to every other cluster within the same model. P values were adjusted for multiple comparisons. Boxes in grey identify non-significant pathway enrichments, whereas boxes in red identify statistically significant enrichments. Bar plot shows percentage of cells identified per cluster in lung lesions.
