# Supplementary material for: Osteosarcoma tumors maintain intra-tumoral transcriptional heterogeneity during bone and lung colonization
Source: BMC Biol. 2023 Apr 27;21:98. doi: 10.1186/s12915-023-01593-3 (PMC10142502; doi:10.1186/s12915-023-01593-3)
Supplement: Supplementary file 8 — Additional file 8: Figure S7. High inter-tumor heterogeneity identified between models. [file 12915_2023_1593_MOESM8_ESM.pdf]

Figure S7

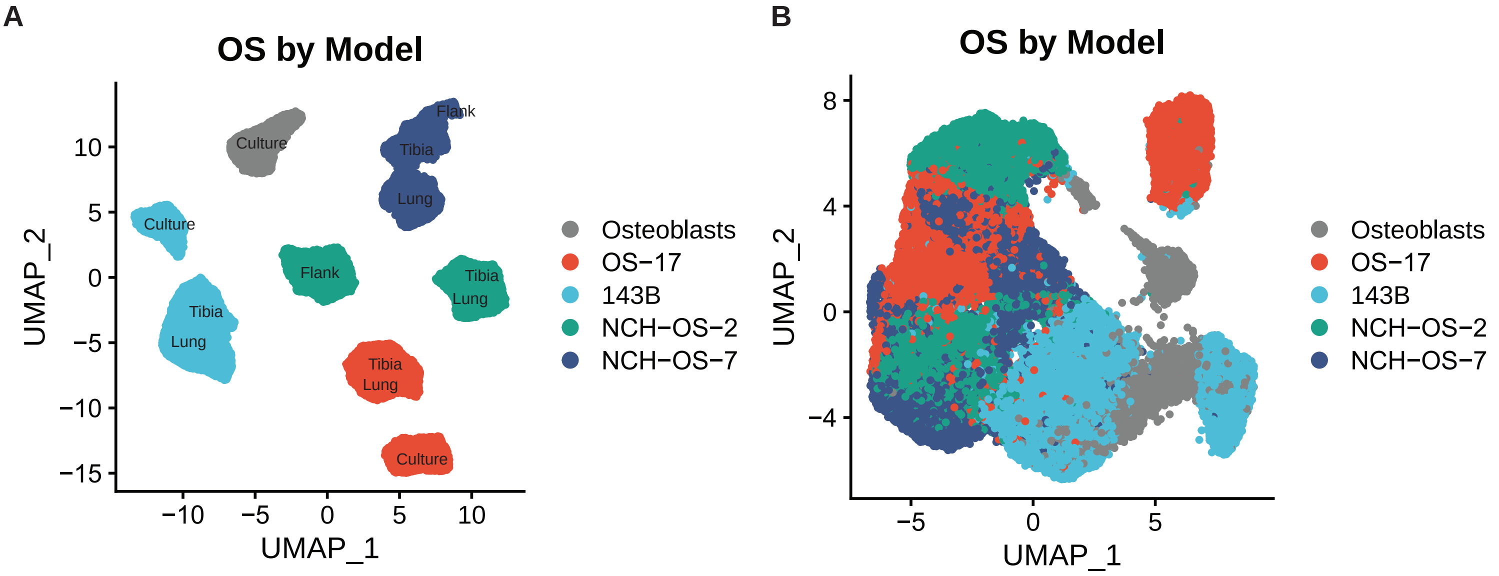

Figure S7. High inter-tumor heterogeneity identified between models. A) UMAP analysis of merged datasets. B) Application of Harmony forces similarity between heterogeneous populations of cells.
