# Supplementary material for: Osteosarcoma tumors maintain intra-tumoral transcriptional heterogeneity during bone and lung colonization
Source: BMC Biol. 2023 Apr 27;21:98. doi: 10.1186/s12915-023-01593-3 (PMC10142502; doi:10.1186/s12915-023-01593-3)
Supplement: Supplementary file 9 — Additional file 9: Figure S8. Integrative analysis for within-model comparison of primary and metastatic lesions. [file 12915_2023_1593_MOESM9_ESM.pdf]

Figure S8

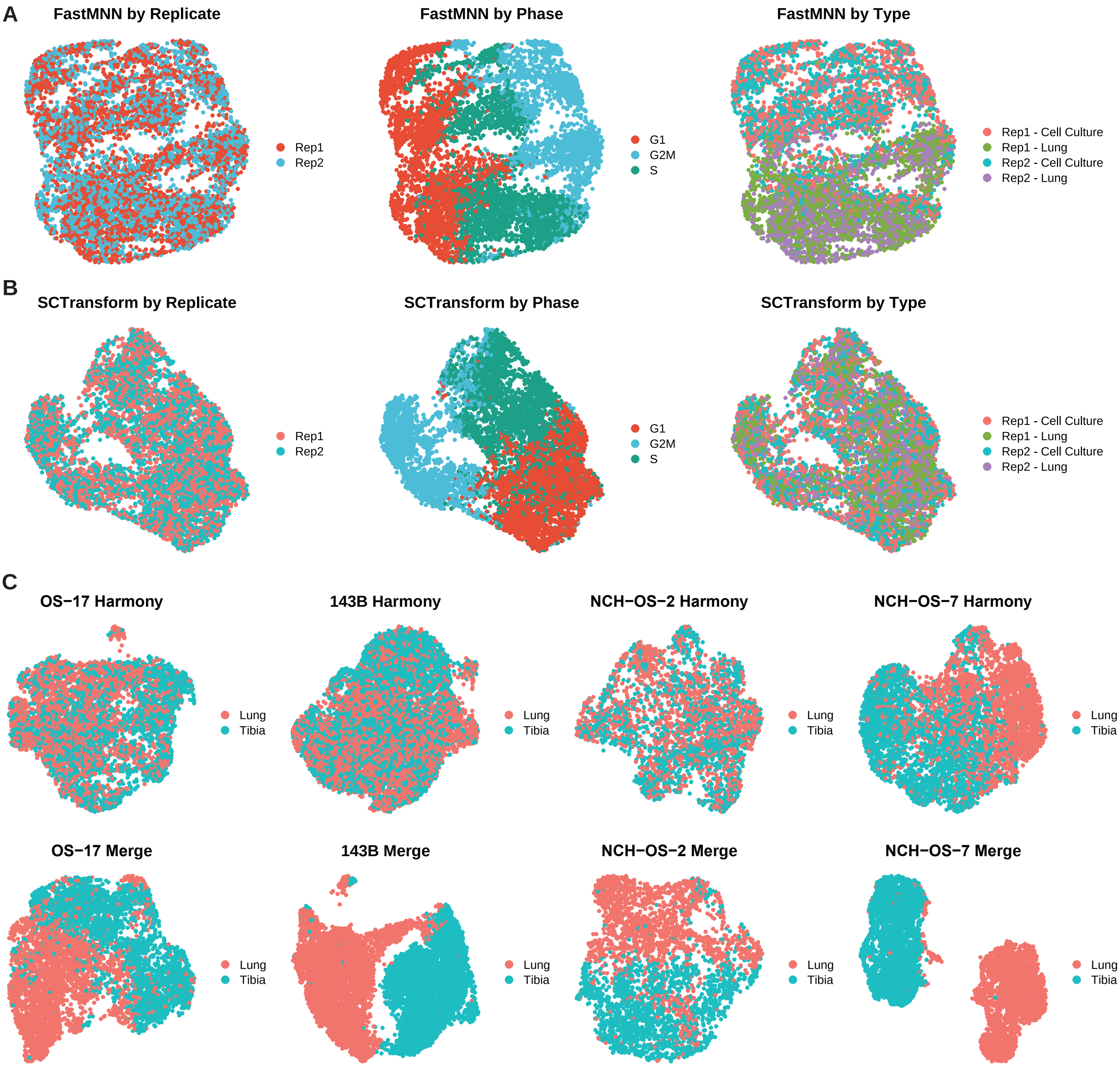

Figure S8. Integrative analysis for within-model comparison of primary and metastatic lesions. A, B) Application of FastMNN or SCTransform between replicate samples demonstrated complete overlay, however cells separated largely by state of cell cycle (OS-17). C) Application of Harmony or merging to Seurat objects. Application of Harmony demonstrated nearly complete overlay. Merged objects displayed some overlay while maintaining a slight distinction between samples. Cells did not cluster based on state of cell cycle (data not shown).
