# Supplementary material for: Osteosarcoma tumors maintain intra-tumoral transcriptional heterogeneity during bone and lung colonization
Source: BMC Biol. 2023 Apr 27;21:98. doi: 10.1186/s12915-023-01593-3 (PMC10142502; doi:10.1186/s12915-023-01593-3)
Supplement: Supplementary file 10 — Additional file 10: Figure S9. Heterogeneous activation of glycolysis identified across multiple cell line and PDX tumor datasets. Figure S10. Heterogeneous activation of hypoxia related genes identified across multiple cell line and PDX tumor datasets. Figure S11. Heterogeneous activation of EMT identified across multiple cell line and PDX tumor datasets. Figure S12. Heterogeneous activation of ‘TNFα signaling via NFκB’ identified across multiple cell line and PDX tumor datasets. [file 12915_2023_1593_MOESM10_ESM.pdf]

Figure S9

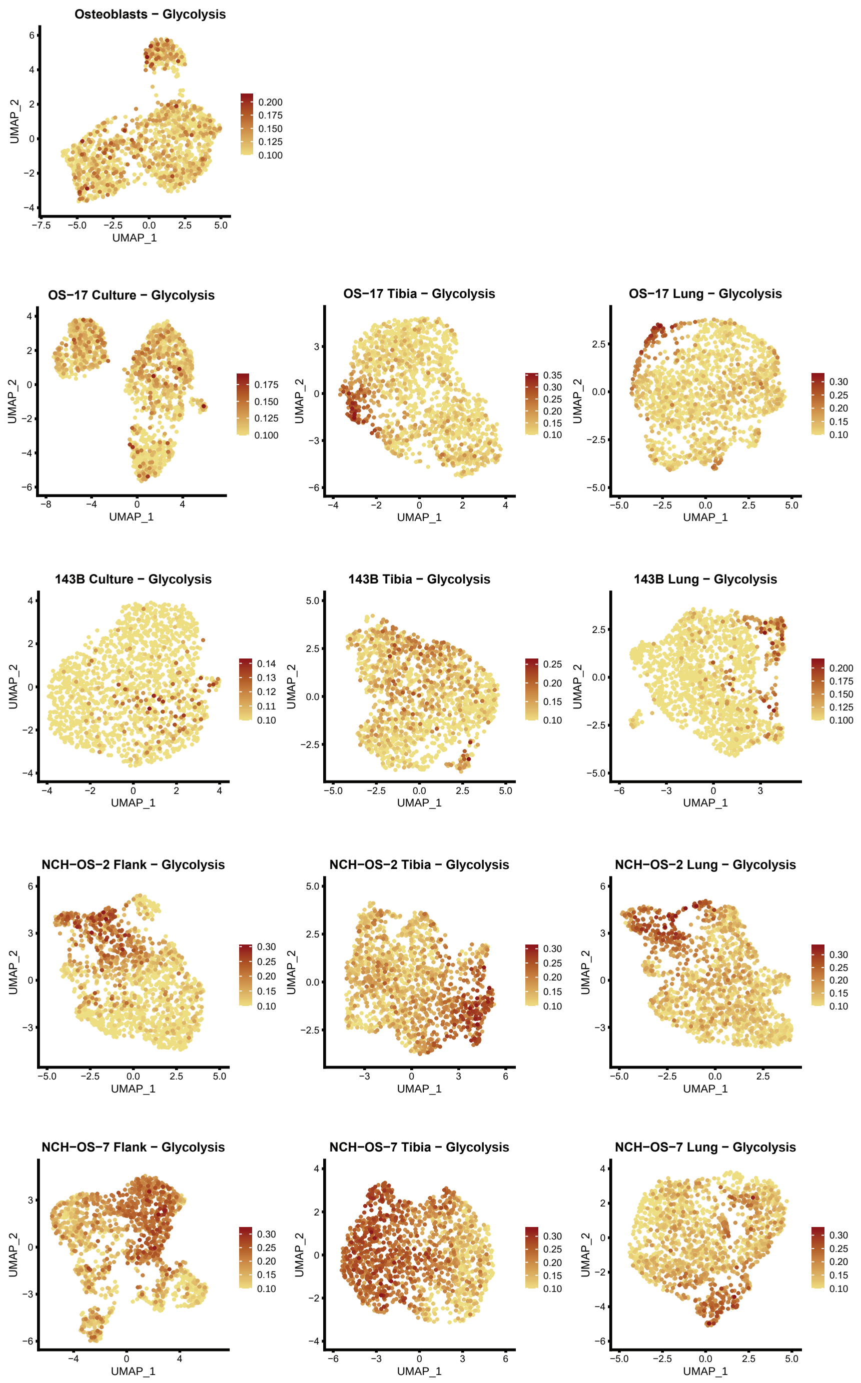

Figure S9. Heterogeneous activation of glycolysis identified across multiple cell line and PDX tumor datasets. FeaturePlots for glycolysis module score in each of the datasets. Module score for glycolysis was calculated using the ‘AddModuleScore’ function in Seurat with msigdb HALLMARK_GLYCOLYSIS genes as input features for the expression program.

Figure S10

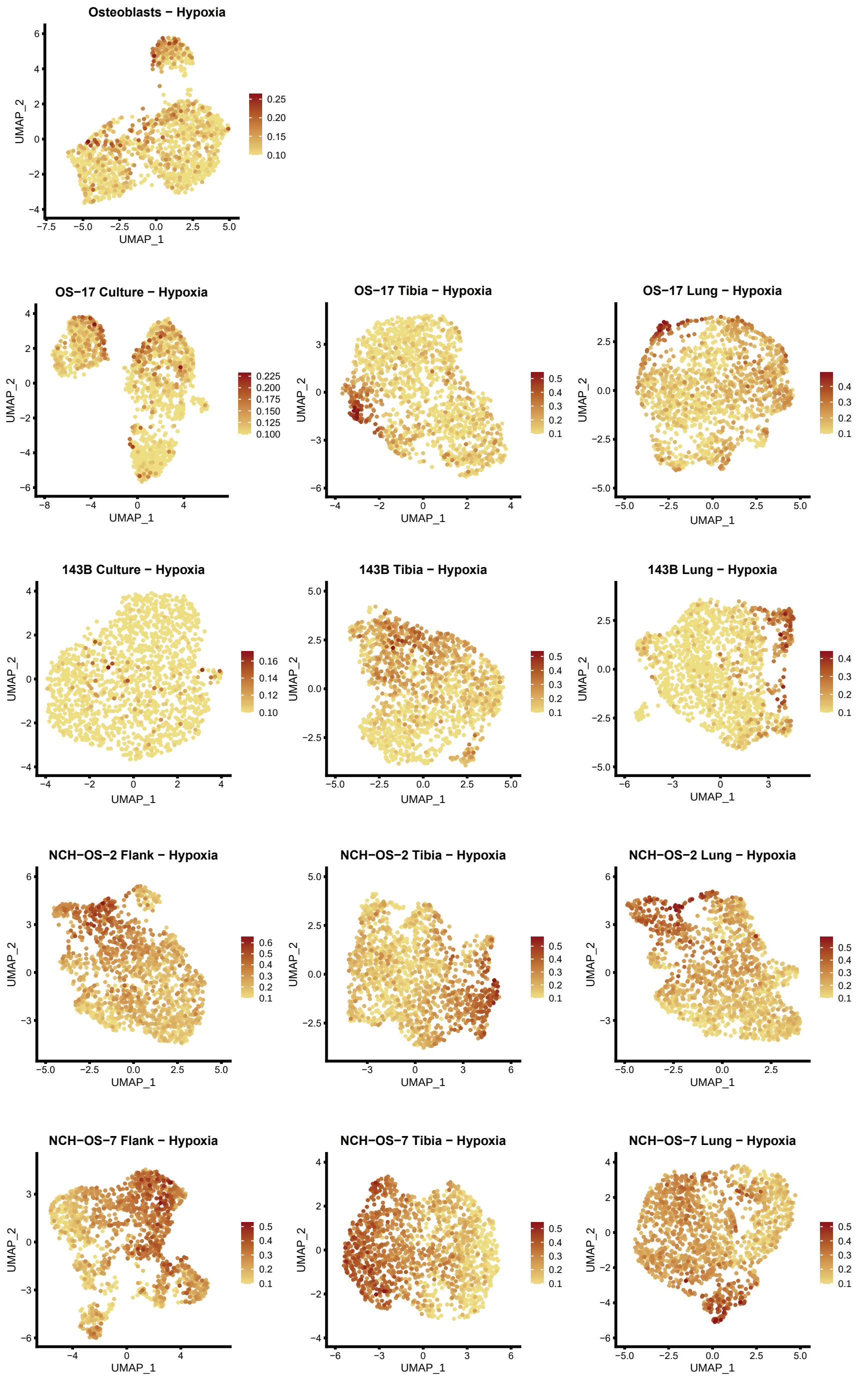

Figure S10. Heterogeneous activation of hypoxia related genes identified across multiple cell line and PDX tumor datasets. FeaturePlots for hypoxia module score in each of the datasets. Module score for hypoxia was calculated using the 'AddModuleScore' function in Seurat with msigdb HALLMARK_HYPOXIA genes as input features for the expression program.

Figure S11

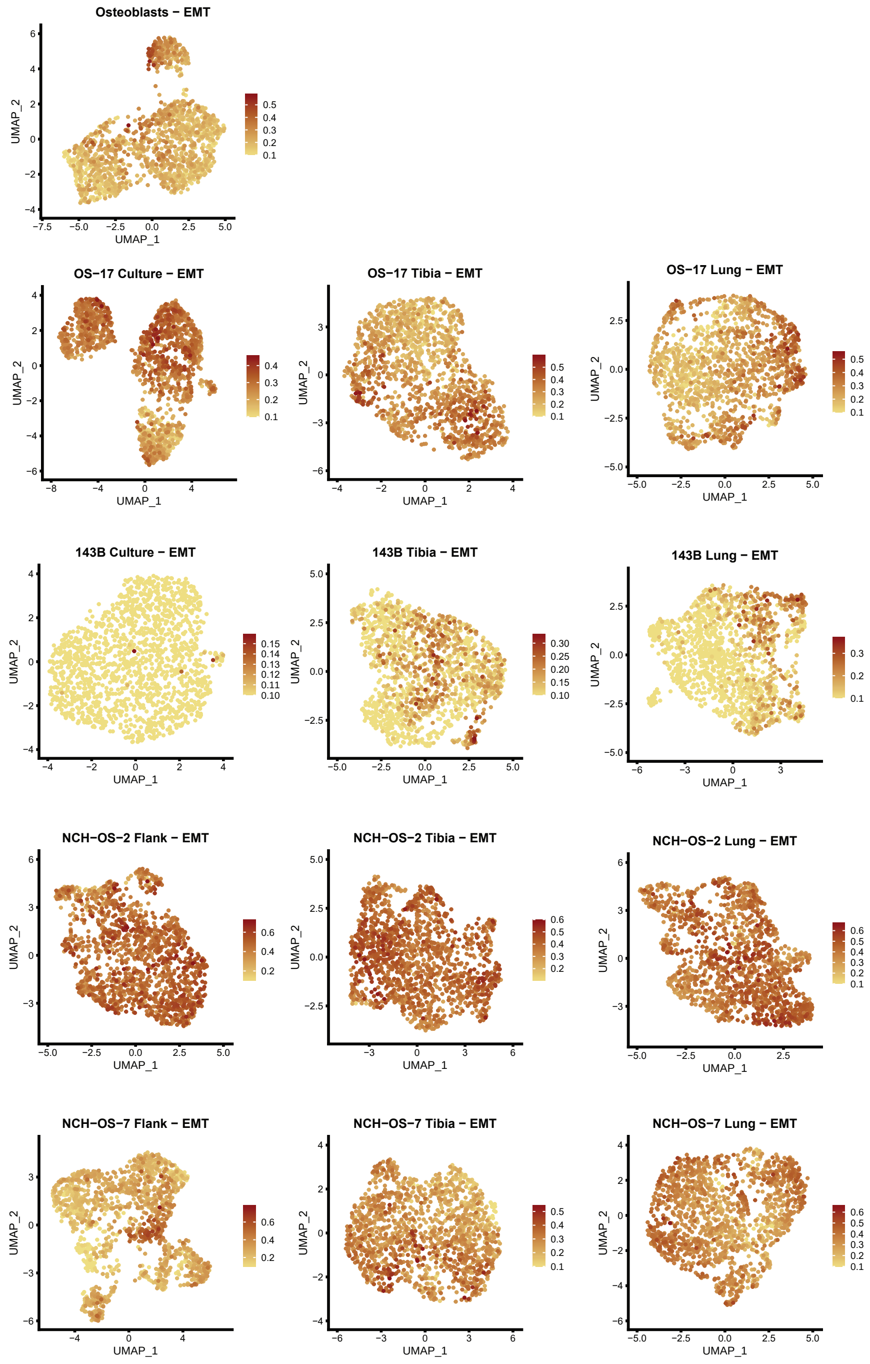

Figure S11. Heterogeneous activation of EMT identified across multiple cell line and PDX tumor datasets. FeaturePlots for EMT module score in each of the datasets. Module score for EMT was calculated using the ‘AddModuleScore’ function in Seurat with msigdb HALLMARK_EMT genes as input features for the expression program.

Figure S12

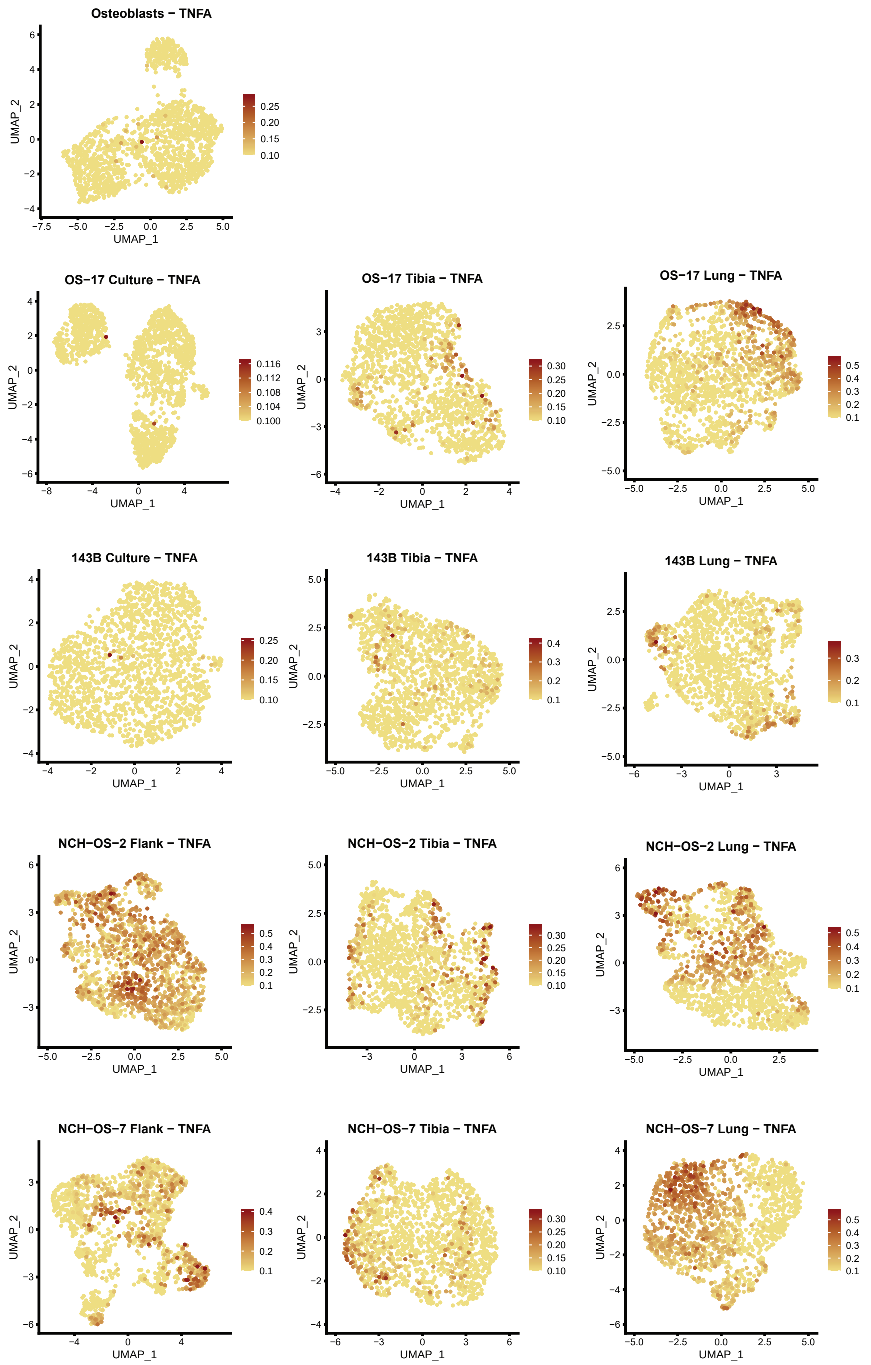

Figure S12. Heterogeneous activation of ‘TNFα signaling via NFκB’ identified across multiple cell line and PDX tumor datasets. FeaturePlots for TNFα signaling via NFκB module score in each of the datasets. Module score for EMT was calculated using the ‘AddModuleScore’ function in Seurat with msigdb HALLMARK_TNFA genes as input features for the expression program.
