# Supplementary material for: Osteosarcoma tumors maintain intra-tumoral transcriptional heterogeneity during bone and lung colonization
Source: BMC Biol. 2023 Apr 27;21:98. doi: 10.1186/s12915-023-01593-3 (PMC10142502; doi:10.1186/s12915-023-01593-3)
Supplement: Supplementary file 11 — Additional file 11: Figure S13. Correlation analysis for multiple cell line and PDX tumor datasets reveals strong positive correlation between glycolysis and hypoxia module scores. Figure S14. Correlation analysis for multiple cell line and PDX tumor datasets reveals minimal correlation between glycolysis and EMT module scores. Figure S15. Correlation analysis for multiple cell line and PDX tumor datasets reveals minimal correlation between glycolysis and ‘TNFα signaling via NFκB’ module scores. Figure S16. Correlation analysis for multiple cell line and PDX tumor datasets reveals mixed correlation between hypoxia and EMT module scores. Figure S17. Correlation analysis for multiple cell line and PDX tumor datasets reveals strong correlation between hypoxia and ‘TNFα signaling via NFκB’ module scores. Figure S18. Correlation analysis for multiple cell line and PDX tumor datasets reveals moderate correlation between EMT and ‘TNFα signaling via NFκB’ module scores. [file 12915_2023_1593_MOESM11_ESM.pdf]

Figure S13

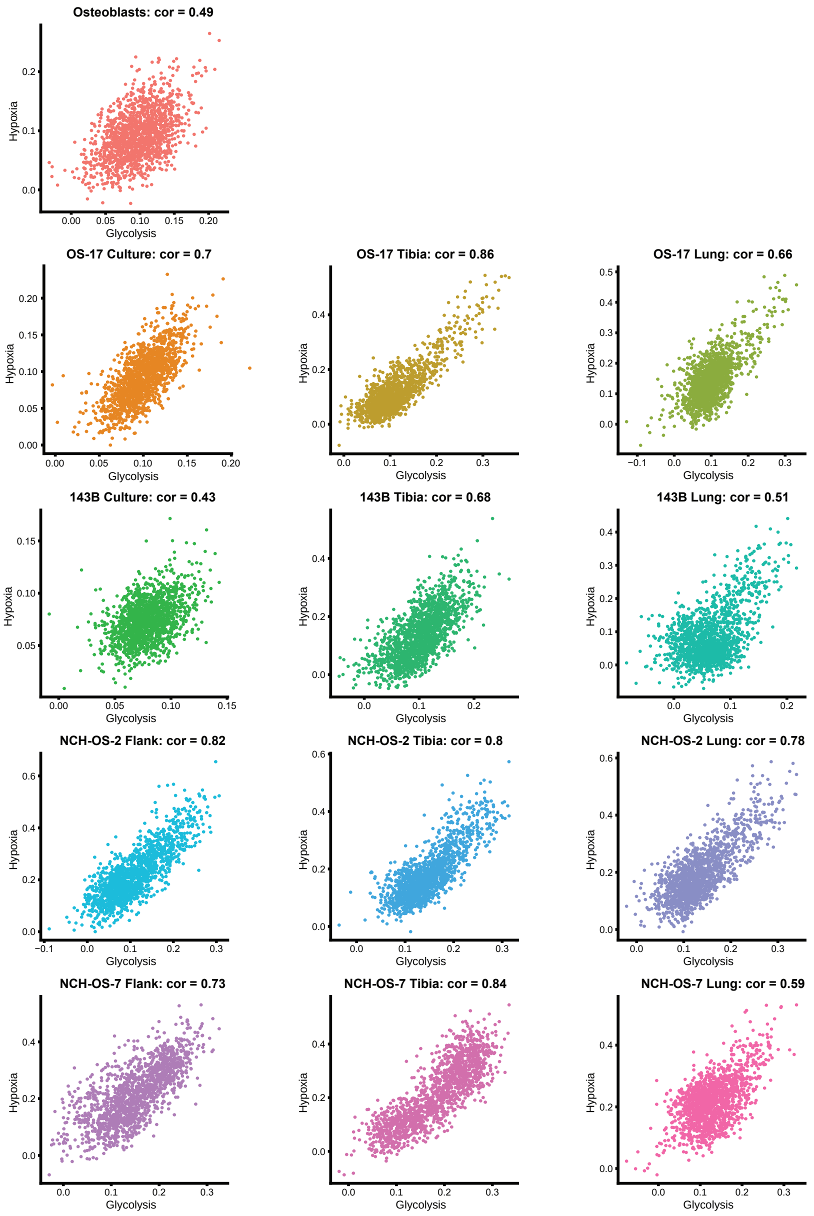


**Figure S13. Correlation analysis for multiple cell line and PDX tumor datasets reveals strong positive correlation between glycolysis and hypoxia module scores.** Scatter plots using the ‘FeatureScatter’ function in Seurat for glycolysis and hypoxia module scores in each of the datasets. Seurat-calculated correlation coefficient for each analysis is shown in the title above each plot.

Figure S14

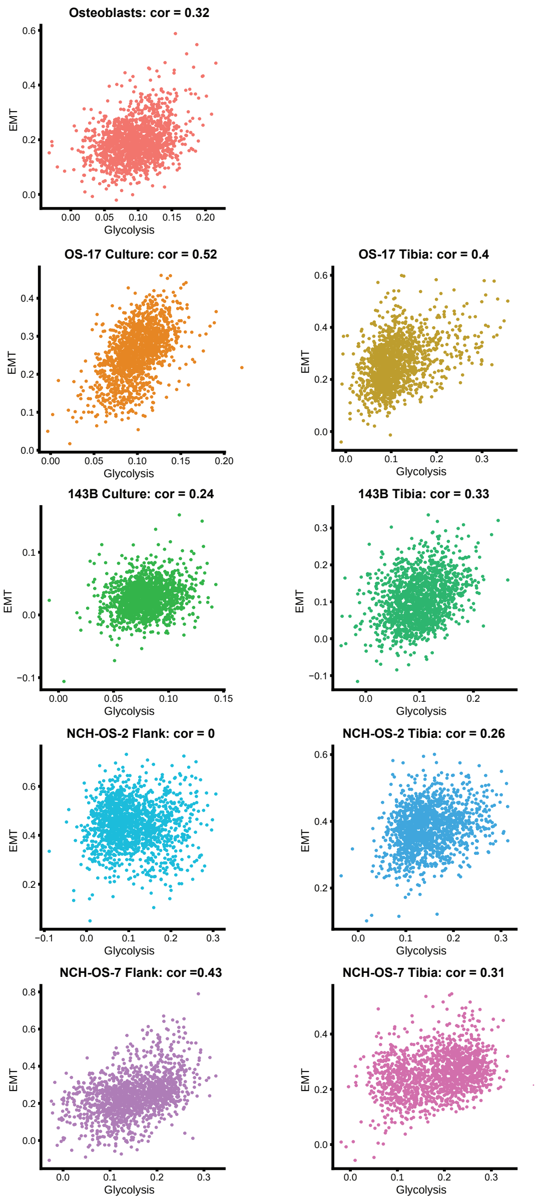


**Figure S14. Correlation analysis for multiple cell line and PDX tumor datasets reveals minimal correlation between glycolysis and EMT module scores.** Scatter plots using the 'FeatureScatter' function in Seurat for glycolysis and EMT module scores in each of the datasets. Seurat-calculated correlation coefficient for each analysis is shown in the title above each plot.

Figure S15

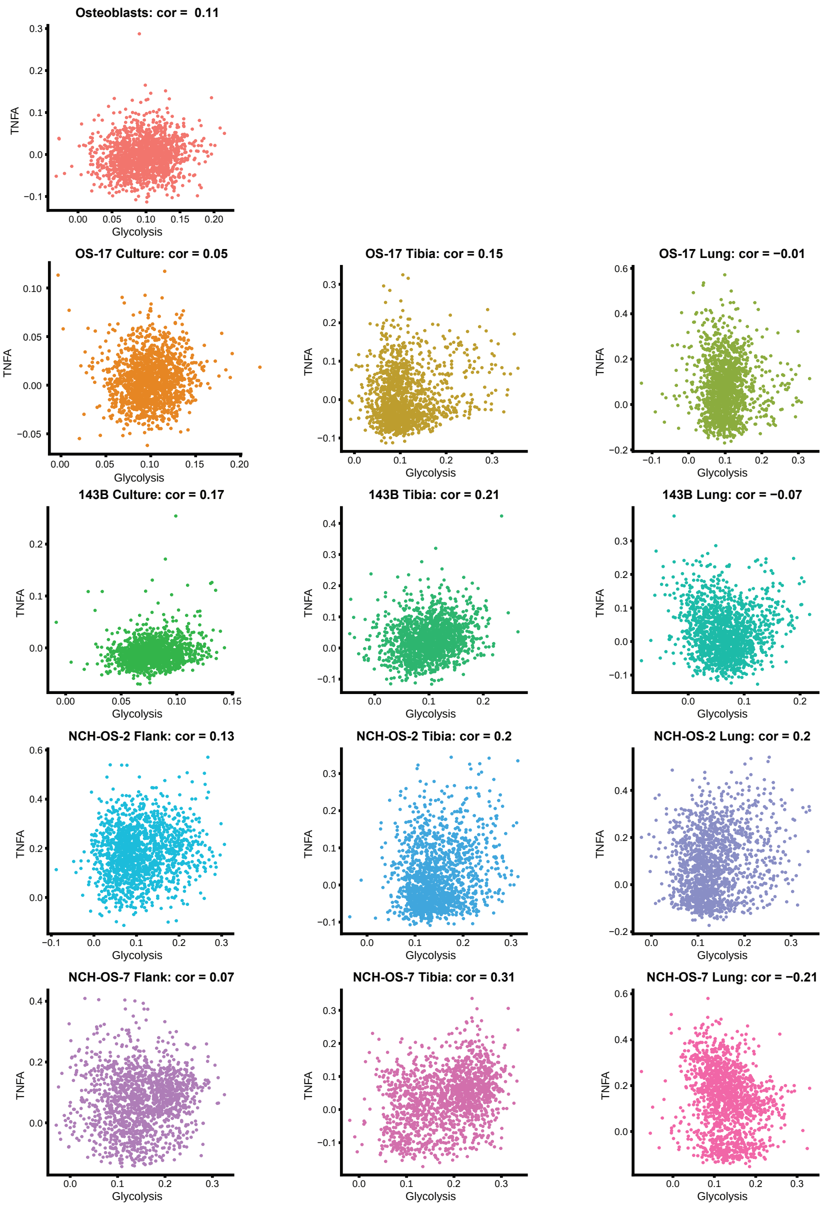


**Figure S15. Correlation analysis for multiple cell line and PDX tumor datasets reveals minimal correlation between glycolysis and 'TNFα signaling via NFκB' module scores.** Scatter plots using the 'FeatureScatter' function in Seurat for glycolysis and TNFα signaling via NFκB module scores in each of the datasets. Seurat-calculated correlation coefficient for each analysis is shown in the title above each plot.

Figure S16

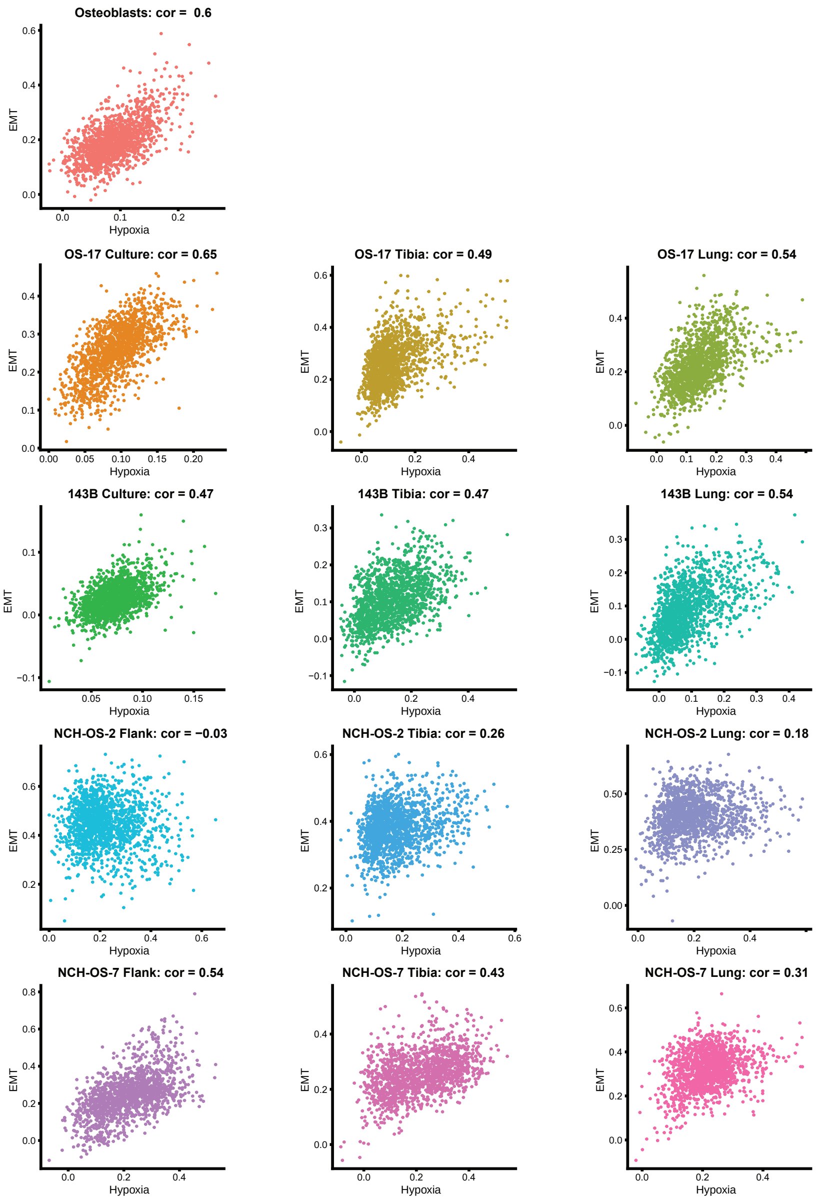


**Figure S16. Correlation analysis for multiple cell line and PDX tumor datasets reveals mixed correlation between hypoxia and EMT module scores.** Scatter plots using the ‘FeatureScatter’ function in Seurat for hypoxia and EMT module scores in each of the datasets. Seurat-calculated correlation coefficient for each analysis is shown in the title above each plot.

Figure S17

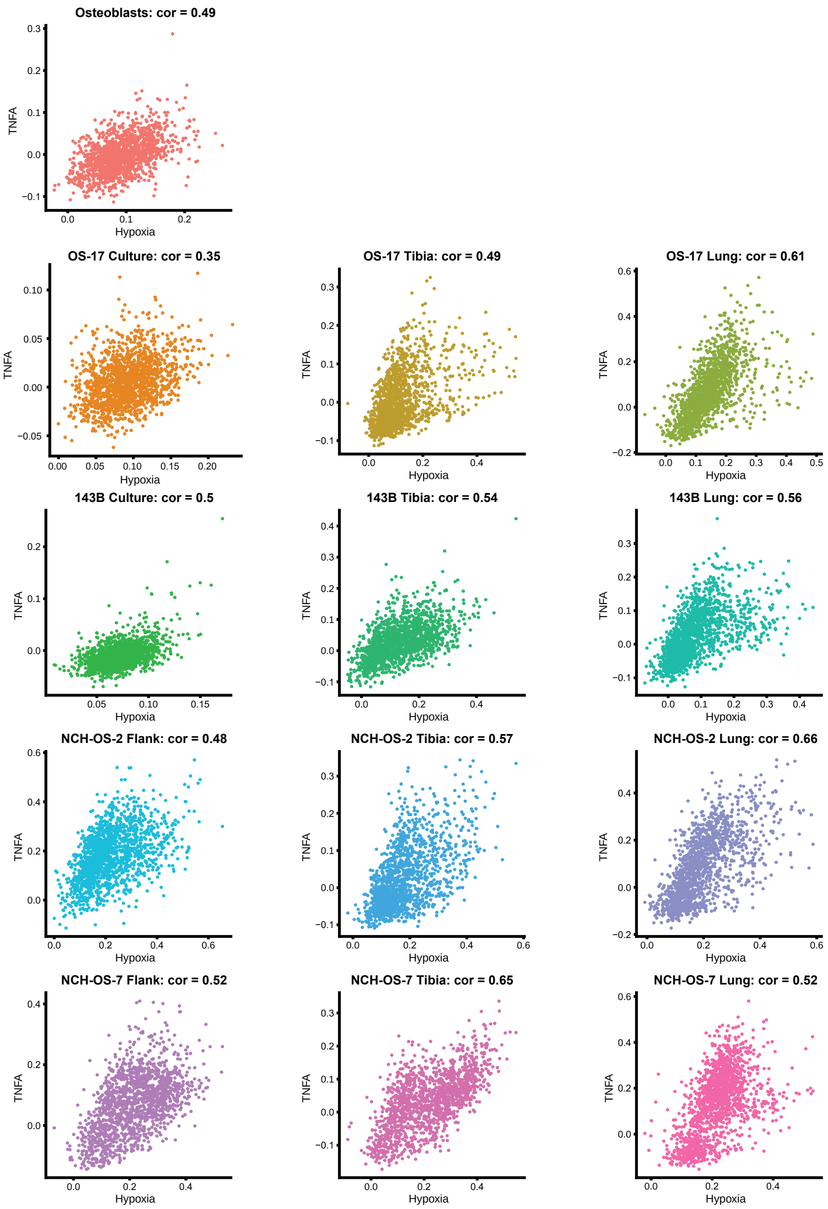


**Figure S17. Correlation analysis for multiple cell line and PDX tumor datasets reveals strong correlation between hypoxia and 'TNFα signaling via NFκB' module scores.** Scatter plots using the 'FeatureScatter' function in Seurat for hypoxia and TNFα signaling via NFκB module scores in each of the datasets. Seurat-calculated correlation coefficient for each analysis is shown in the title above each plot.

Figure S18

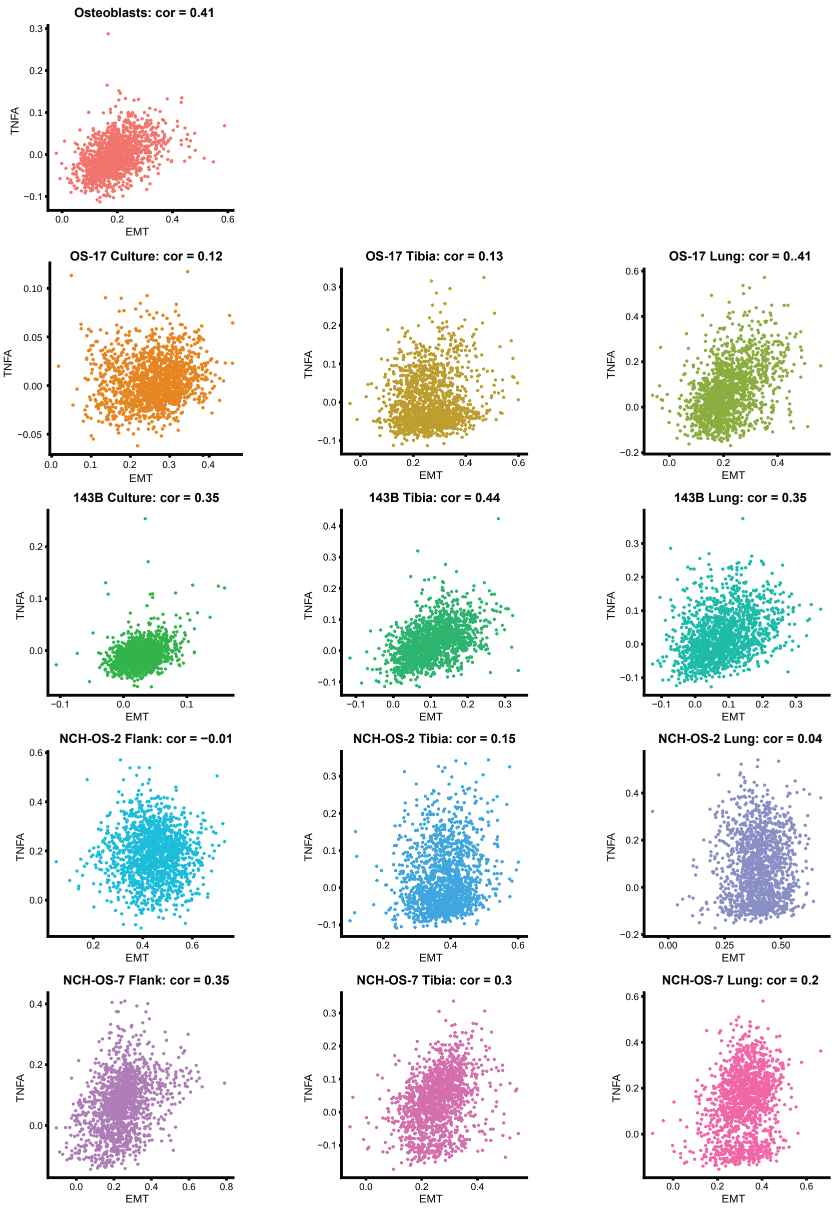


**Figure S18. Correlation analysis for multiple cell line and PDX tumor datasets reveals moderate correlation between EMT and 'TNFα signaling via NFκB' module scores.** Scatter plots using the 'FeatureScatter' function in Seurat for EMT and TNFα signaling via NFκB module scores in each of the datasets. Seurat-calculated correlation coefficient for each analysis is shown in the title above each plot.
