# Supplementary material for: Osteosarcoma tumors maintain intra-tumoral transcriptional heterogeneity during bone and lung colonization
Source: BMC Biol. 2023 Apr 27;21:98. doi: 10.1186/s12915-023-01593-3 (PMC10142502; doi:10.1186/s12915-023-01593-3)
Supplement: Supplementary file 12 — Additional file 12: Figure S19. Heterogeneous activation of glycolysis identified across multiple patient primary and metastatic tumor datasets. Figure S20. Heterogeneous activation of hypoxia related genes identified across multiple patient primary and metastatic tumor datasets. Figure S21. Heterogeneous activation of EMT related genes identified across multiple patient primary and metastatic tumor datasets. Figure S22. Heterogeneous activation of ‘TNFα signaling via NFκB’ related genes identified across multiple patient primary and metastatic tumor datasets. [file 12915_2023_1593_MOESM12_ESM.pdf]

Figure S19

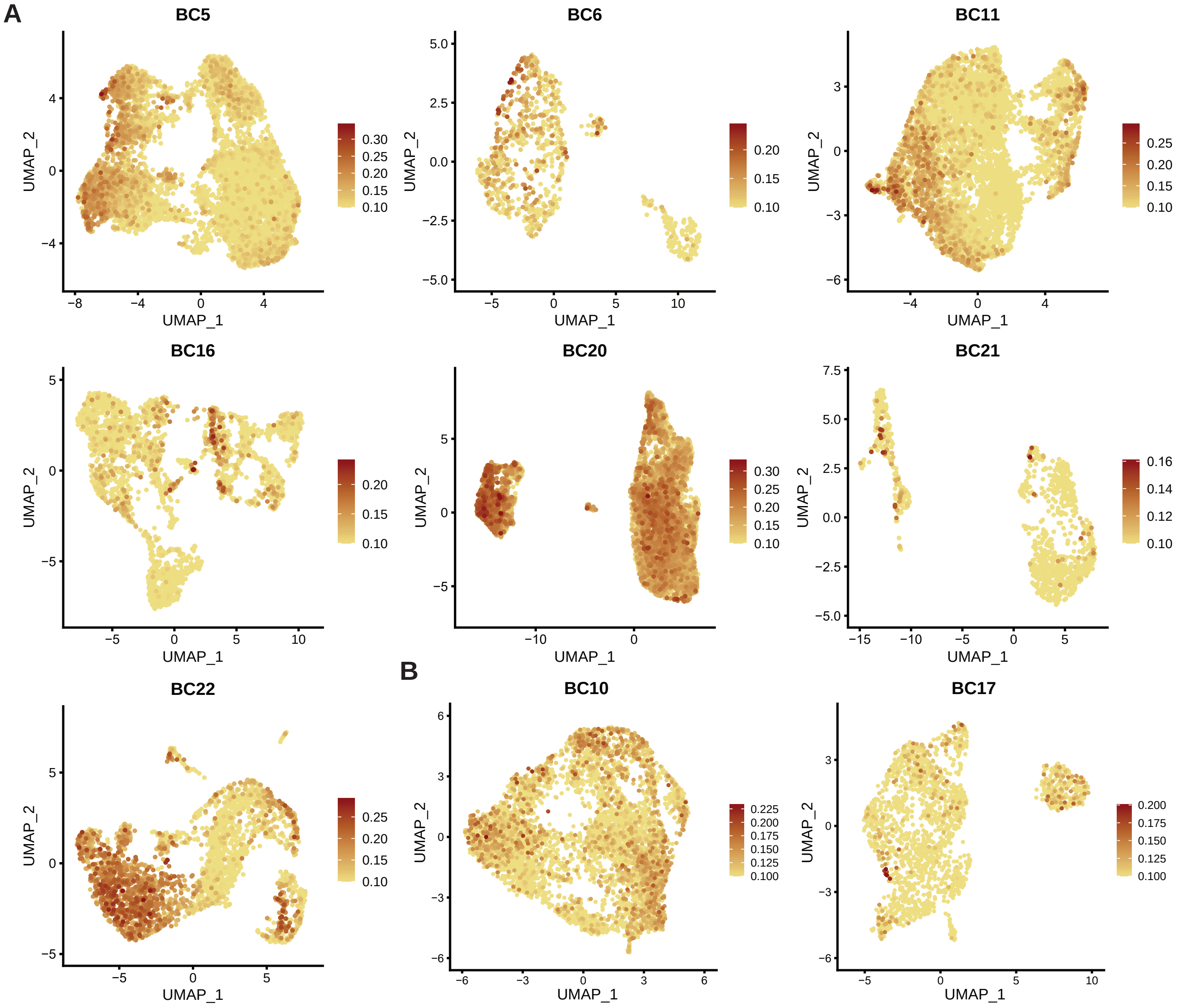

Figure S19. Heterogeneous activation of glycolysis identified across multiple patient primary and metastatic tumor datasets. A) Primary and B) metastatic patient tumor FeaturePlots showing glycolysis module score in each of the datasets. Module score for glycolysis was calculated using the ‘AddModuleScore’ function in Seurat with msigdb HALLMARK_GLYCOLYSIS genes as input features for the expression program. Patient data is from GSE152048.

Figure S20

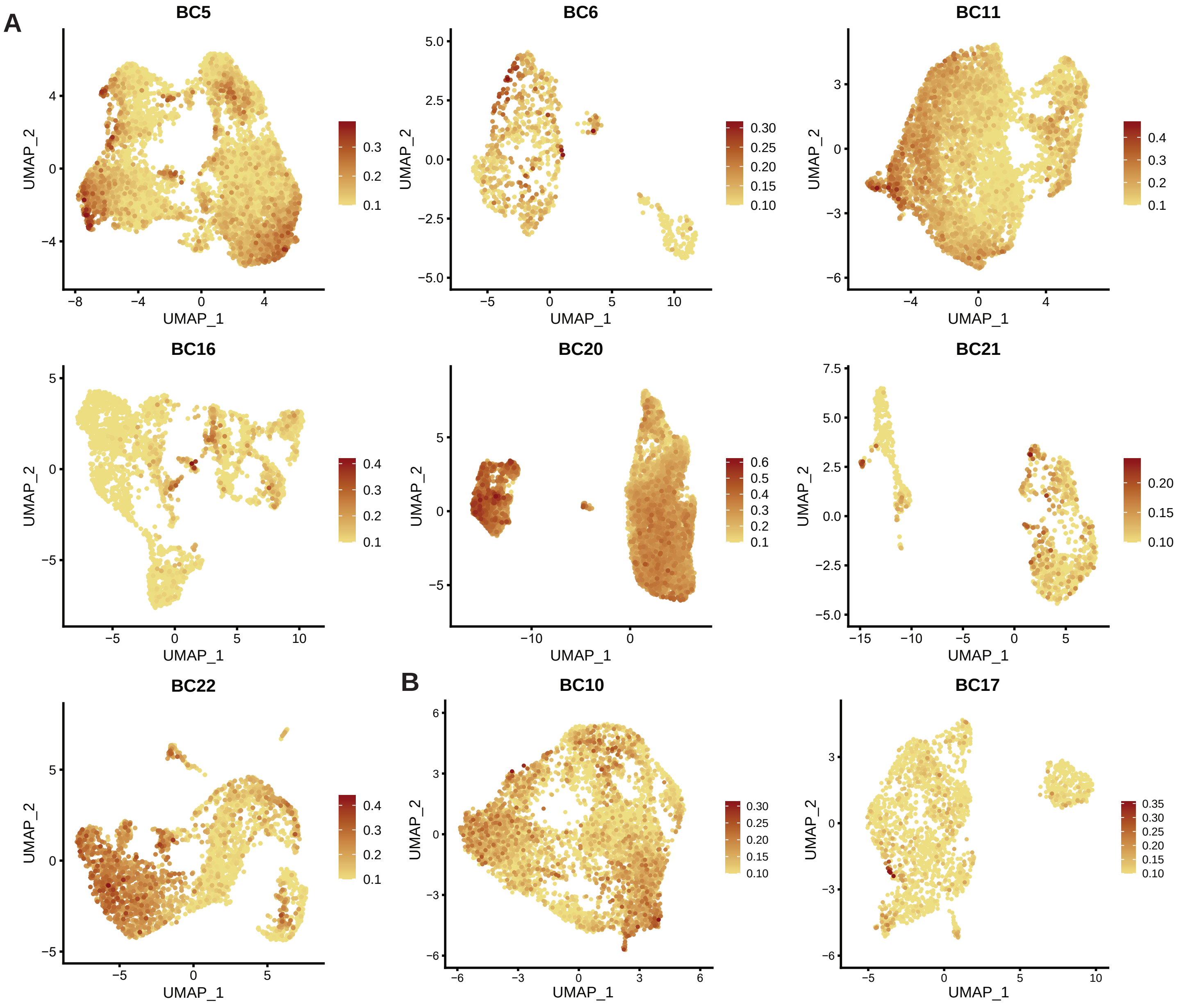

Figure S20. Heterogeneous activation of hypoxia related genes identified across multiple patient primary and metastatic tumor datasets. A) Primary and B) metastatic patient tumor FeaturePlots showing hypoxia module score in each of the datasets. Module score for hypoxia was calculated using the 'AddModule-Score' function in Seurat with msigdb HALLMARK_HYPOXIA genes as input features for the expression program. Patient data is from GSE152048.

Figure S21

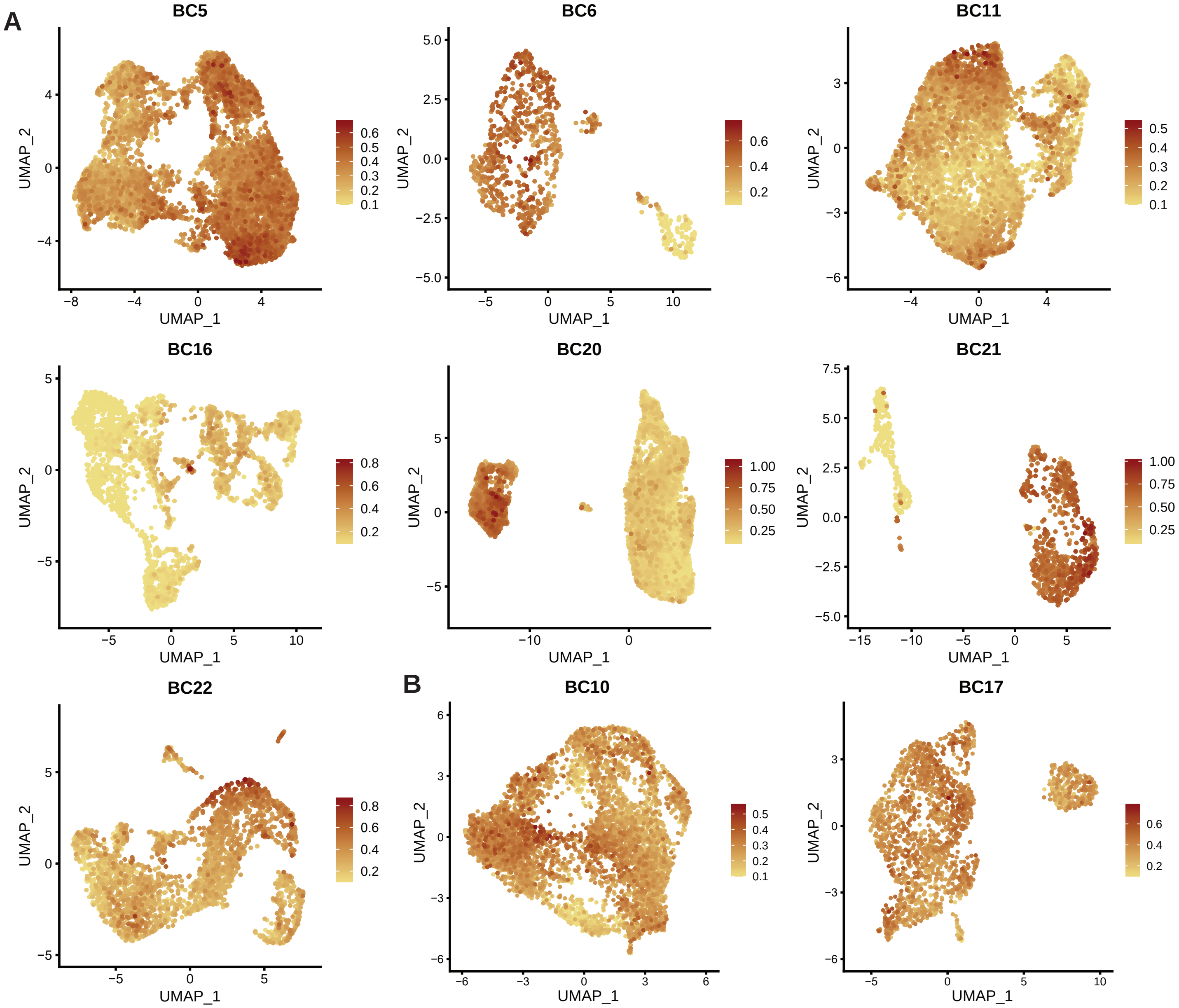

Figure S21. Heterogeneous activation of EMT related genes identified across multiple patient primary and metastatic tumor datasets. A) Primary and B) metastatic patient tumor FeaturePlots showing EMT module score in each of the datasets. Module score for EMT was calculated using the 'AddModuleScore' function in Seurat with msigdb HALLMARK_EPITHELIAL_MESENCHYMAL_TRANSITION genes as input features for the expression program. Patient data is from GSE152048.

Figure S22

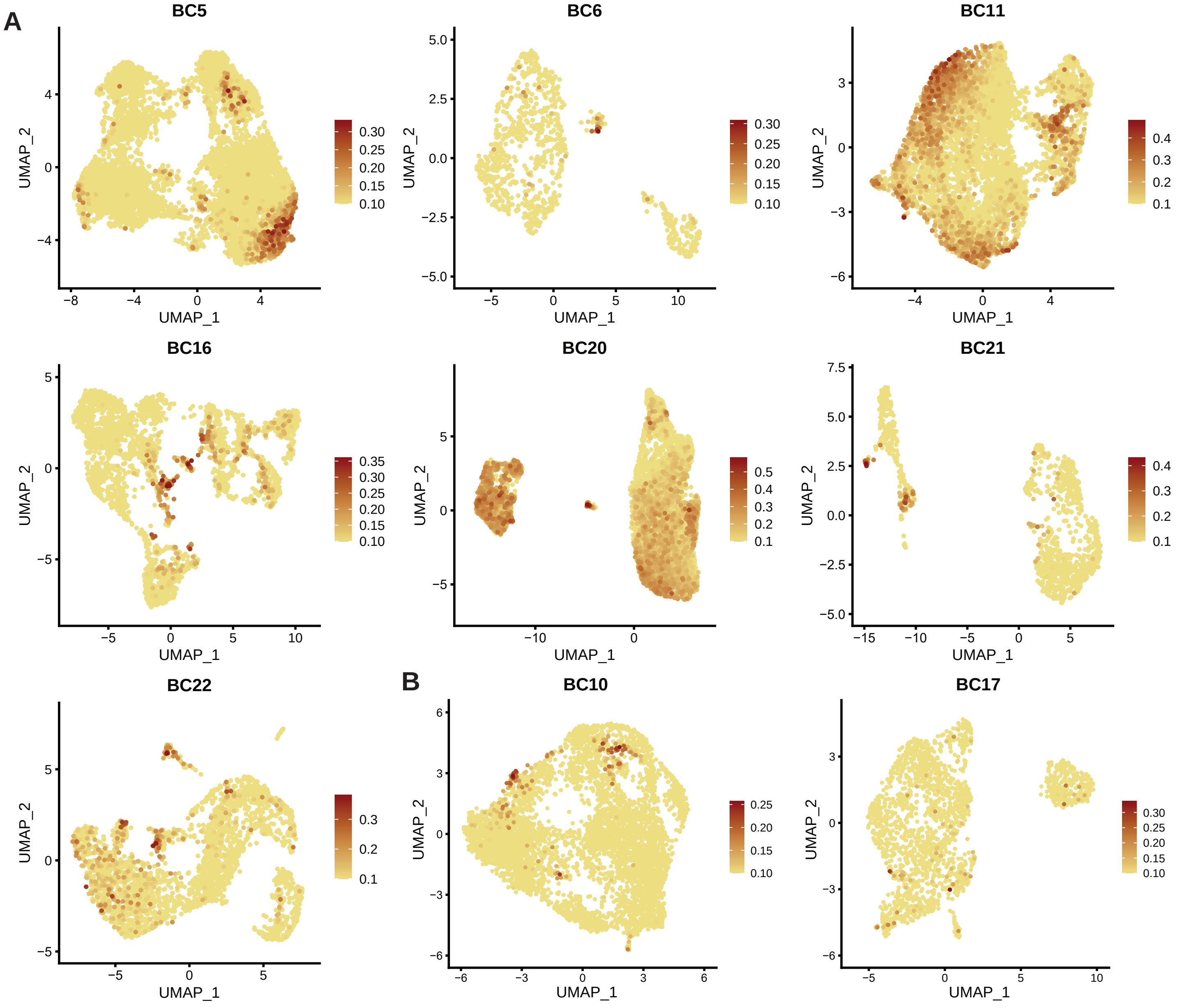

Figure S22. Heterogeneous activation of ‘TNFα signaling via NFκB’ related genes identified across multiple patient primary and metastatic tumor datasets. A) Primary and B) metastatic patient tumor FeaturePlots showing TNFα signaling via NFκB module score in each of the datasets. Module score for TNFα signaling via NFκB was calculated using the ‘AddModuleScore’ function in Seurat with msigdb HALLMARK_TNFA_SIGNALING_VIA_NFKB genes as input features for the expression program. Patient data is from GSE152048.
