# Supplementary material for: Osteosarcoma tumors maintain intra-tumoral transcriptional heterogeneity during bone and lung colonization
Source: BMC Biol. 2023 Apr 27;21:98. doi: 10.1186/s12915-023-01593-3 (PMC10142502; doi:10.1186/s12915-023-01593-3)
Supplement: Supplementary file 13 — Additional file 13: Figure S23. Correlation analysis for multiple patient primary and metastatic tumor datasets reveals strong correlation between glycolysis and hypoxia module scores. Figure S24. Correlation analysis for multiple patient primary and metastatic tumor datasets reveals minimal correlation between glycolysis and EMT module scores. Figure S25. Correlation analysis for multiple patient primary and metastatic tumor datasets reveals minimal correlation between glycolysis and ‘TNFα signaling via NFκB’ module scores. Figure S26. Correlation analysis for multiple patient primary and metastatic tumor datasets reveals mixed correlation between hypoxia and EMT module scores. Figure S27. Correlation analysis for multiple patient primary and metastatic tumor datasets reveals strong correlation between hypoxia and ‘TNFα signaling via NFκB’ module scores. Figure S28. Correlation analysis for multiple patient primary and metastatic tumor datasets reveals moderate correlation between EMT and ‘TNFα signaling via NFκB’ module scores. [file 12915_2023_1593_MOESM13_ESM.pdf]

Figure S23

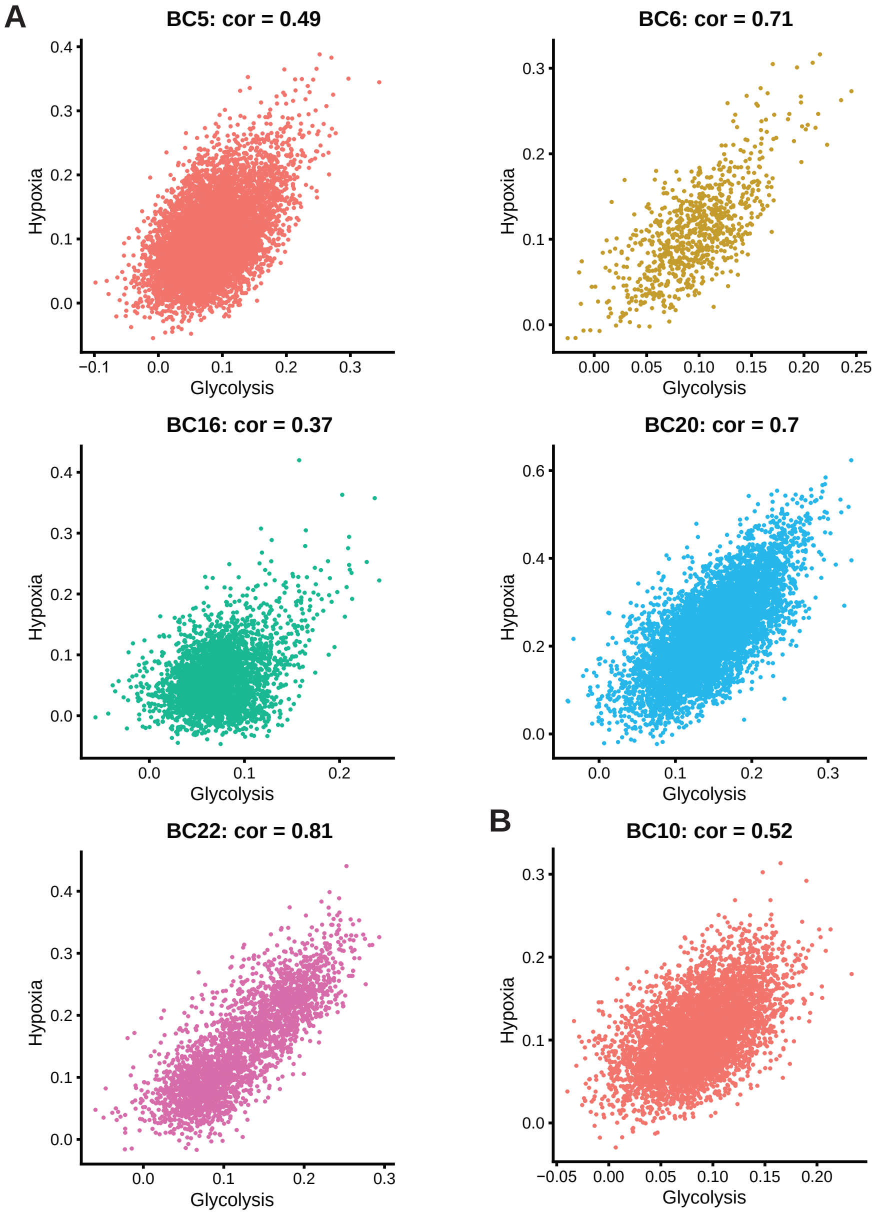

Figure S23. Correlation analysis for multiple patient primary and metastatic tumor datasets reveals strong correlation between glycolysis and hypoxia module scores. A) Primary and B) metastatic patient tumor scatter plots using the ‘FeatureScatter’ function in Seurat for glycolysis and hypoxia module scores in each of the datasets. Seurat-calculated correlation coefficient for each analysis is shown in the title above each plot. Patient data is from GSE152048.

Figure S24

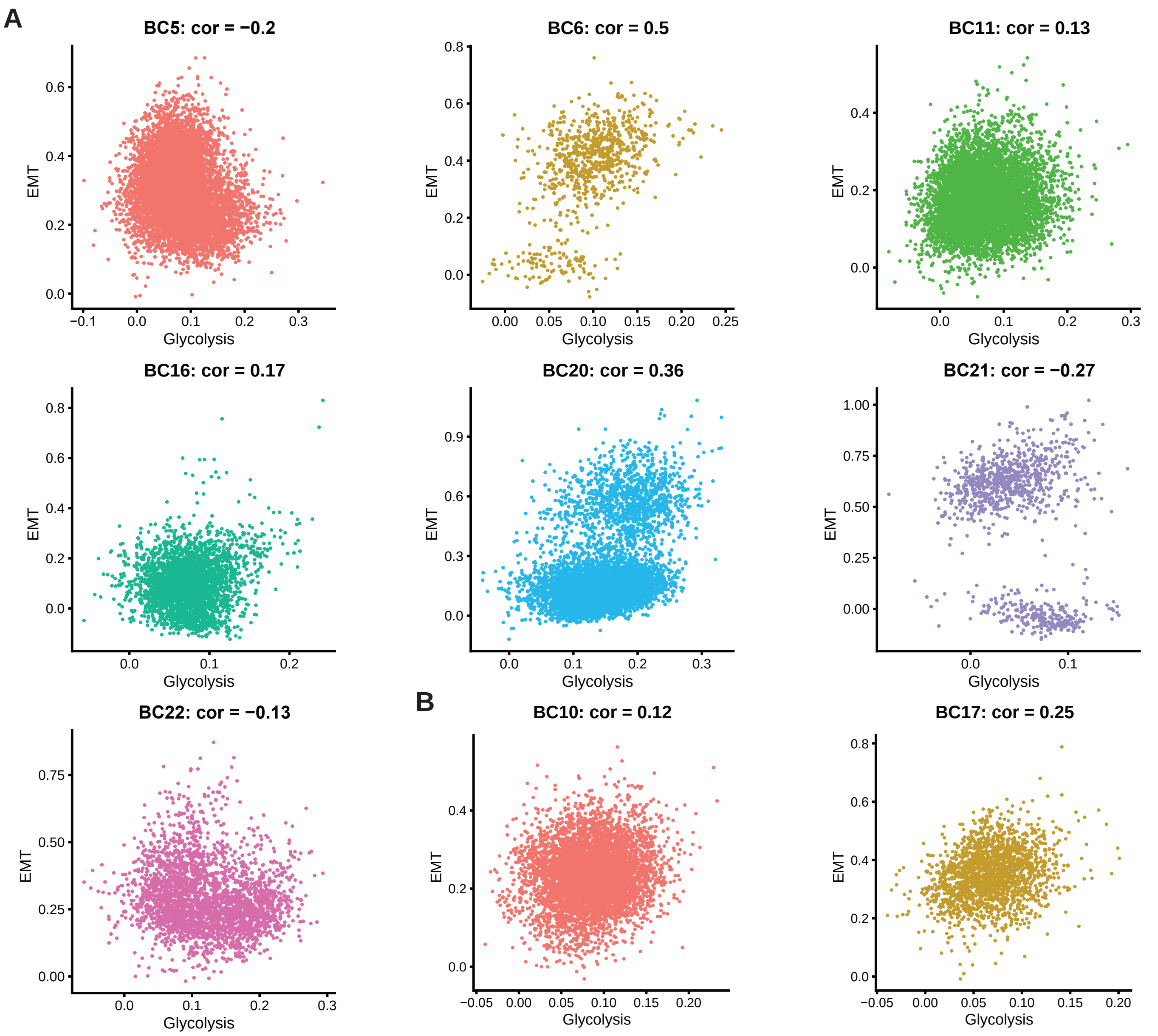

Figure S24. Correlation analysis for multiple patient primary and metastatic tumor datasets reveals minimal correlation between glycolysis and EMT module scores. A) Primary and B) metastatic patient tumor scatter plots using the 'FeatureScatter' function in Seurat for glycolysis and EMT module scores in each of the datasets. Seurat-calculated correlation coefficient for each analysis is shown in the title above each plot. Patient data is from GSE152048.

Figure S25

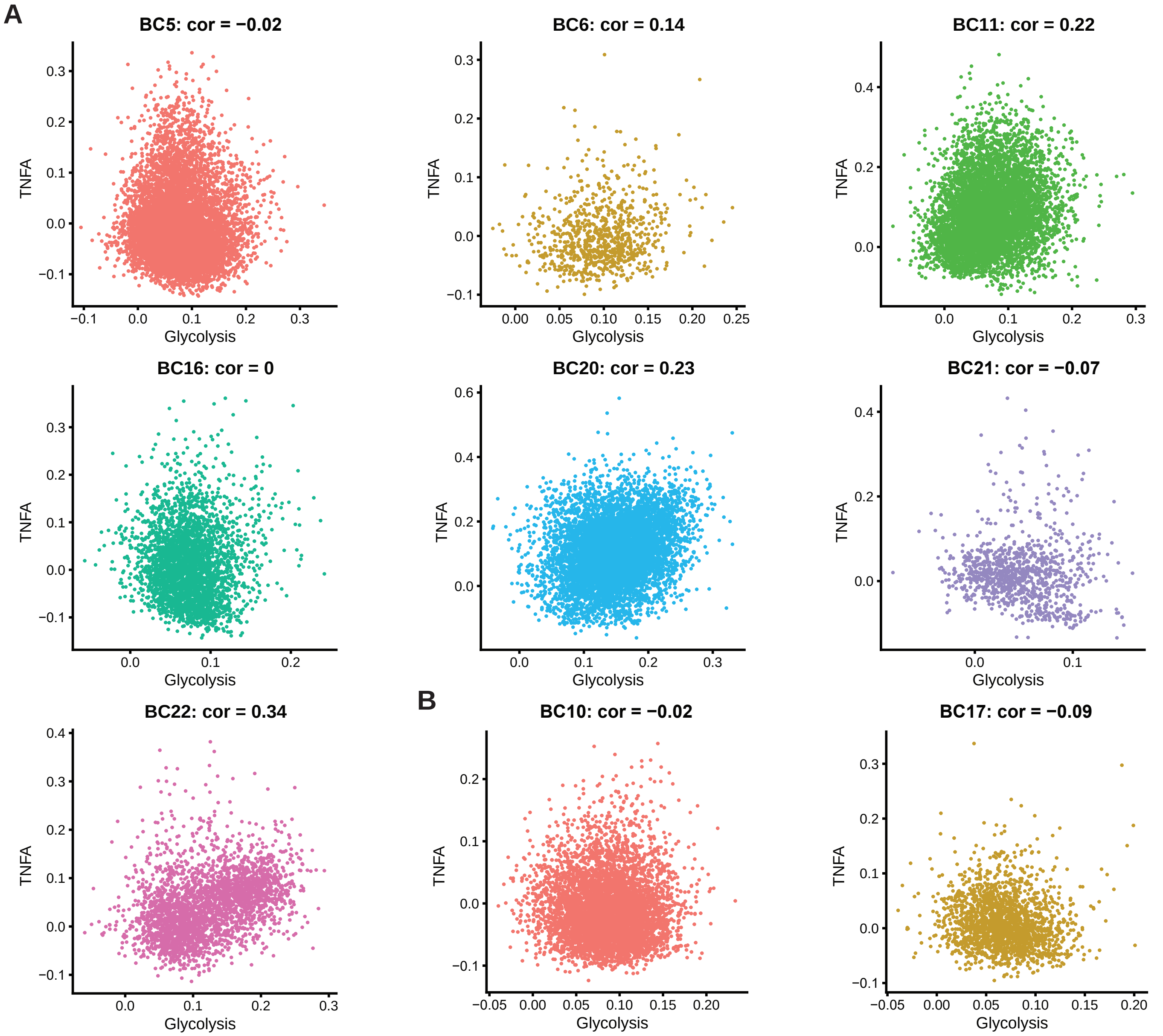

Figure S25. Correlation analysis for multiple patient primary and metastatic tumor datasets reveals minimal correlation between glycolysis and ‘TNFα signaling via NFκB’ module scores. A) Primary and B) metastatic patient tumor scatter plots using the ‘FeatureScatter’ function in Seurat for glycolysis and TNFα signaling via NFκB module scores in each of the datasets. Seurat-calculated correlation coefficient for each analysis is shown in the title above each plot. Patient data is from GSE152048.

Figure S26

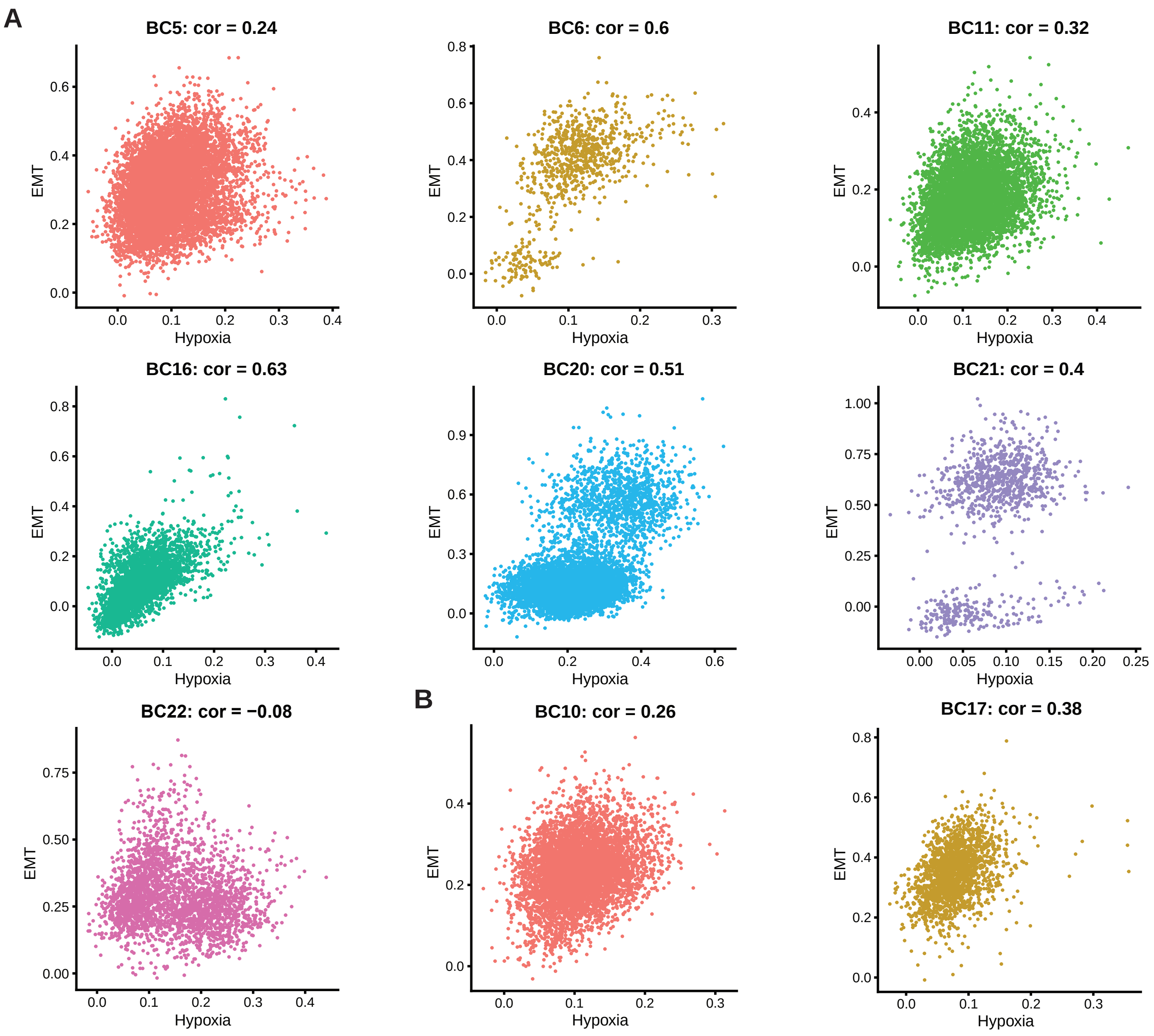

Figure S26. Correlation analysis for multiple patient primary and metastatic tumor datasets reveals mixed correlation between hypoxia and EMT module scores. A) Primary and B) metastatic patient tumor scatter plots using the 'FeatureScatter' function in Seurat for hypoxia and EMT module scores in each of the datasets. Seurat-calculated correlation coefficient for each analysis is shown in the title above each plot. Patient data is from GSE152048.

Figure S27

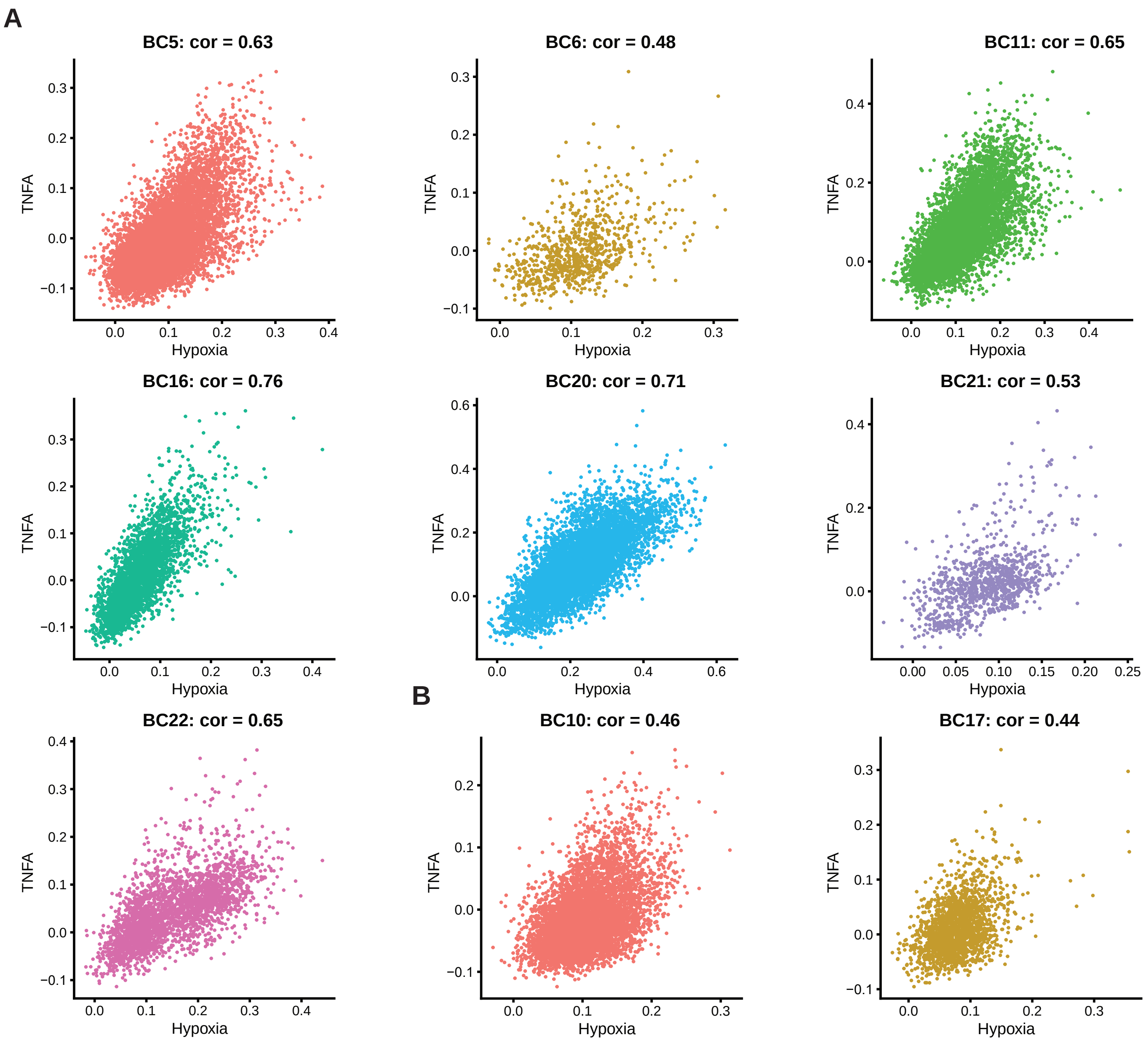

Figure S27. Correlation analysis for multiple patient primary and metastatic tumor datasets reveals strong correlation between hypoxia and 'TNFα signaling via NFκB' module scores. A) Primary and B) metastatic patient tumor scatter plots using the 'FeatureScatter' function in Seurat for hypoxia and TNFα signaling via NFκB module scores in each of the datasets. Seurat-calculated correlation coefficient for each analysis is shown in the title above each plot. Patient data is from GSE152048.

Figure S28

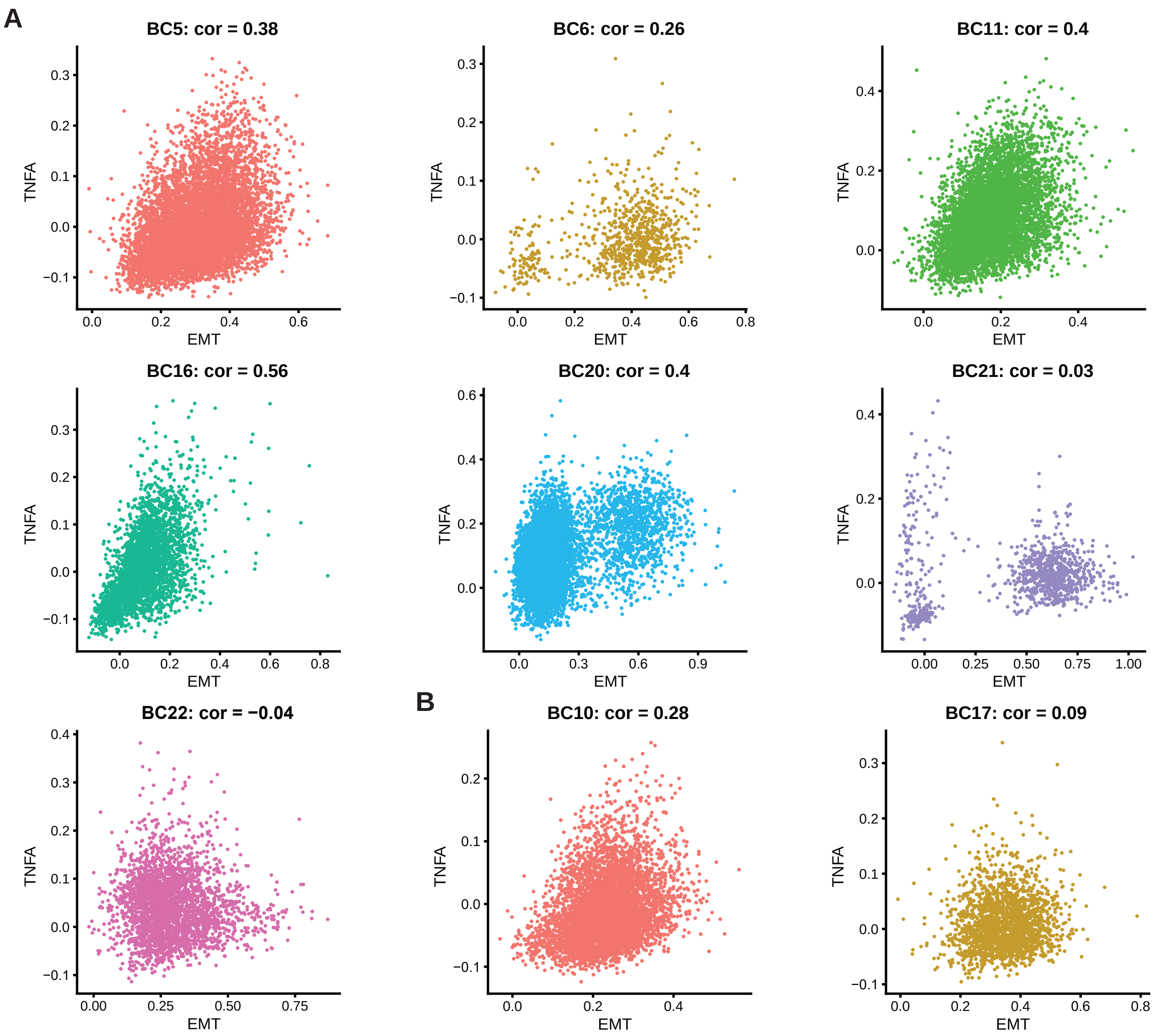

Figure S28. Correlation analysis for multiple patient primary and metastatic tumor datasets reveals moderate correlation between EMT and 'TNFα signaling via NFκB' module scores. A) Primary and B) metastatic patient tumor scatter plots using the 'FeatureScatter' function in Seurat for EMT and TNFα signaling via NFκB module scores in each of the datasets. Seurat-calculated correlation coefficient for each analysis is shown in the title above each plot. Patient data is from GSE152048.
