# Supplementary material for: Osteosarcoma tumors maintain intra-tumoral transcriptional heterogeneity during bone and lung colonization
Source: BMC Biol. 2023 Apr 27;21:98. doi: 10.1186/s12915-023-01593-3 (PMC10142502; doi:10.1186/s12915-023-01593-3)
Supplement: Supplementary file 14 — Additional file 14: Figure S29. High correlation between multiple markers of glycolysis activation in primary and metastatic osteosarcoma lesions. [file 12915_2023_1593_MOESM14_ESM.pdf]

Figure S29

A

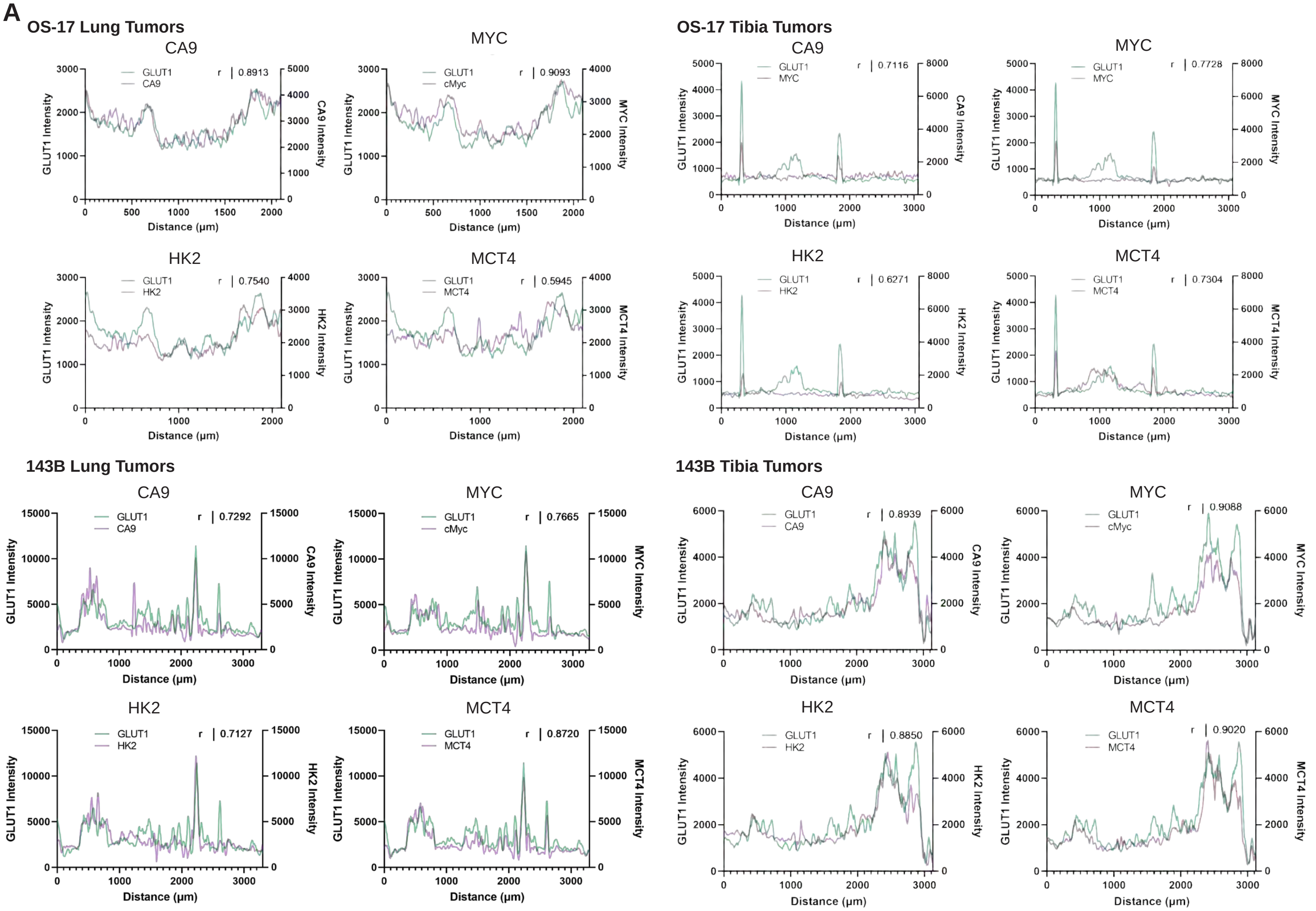

B

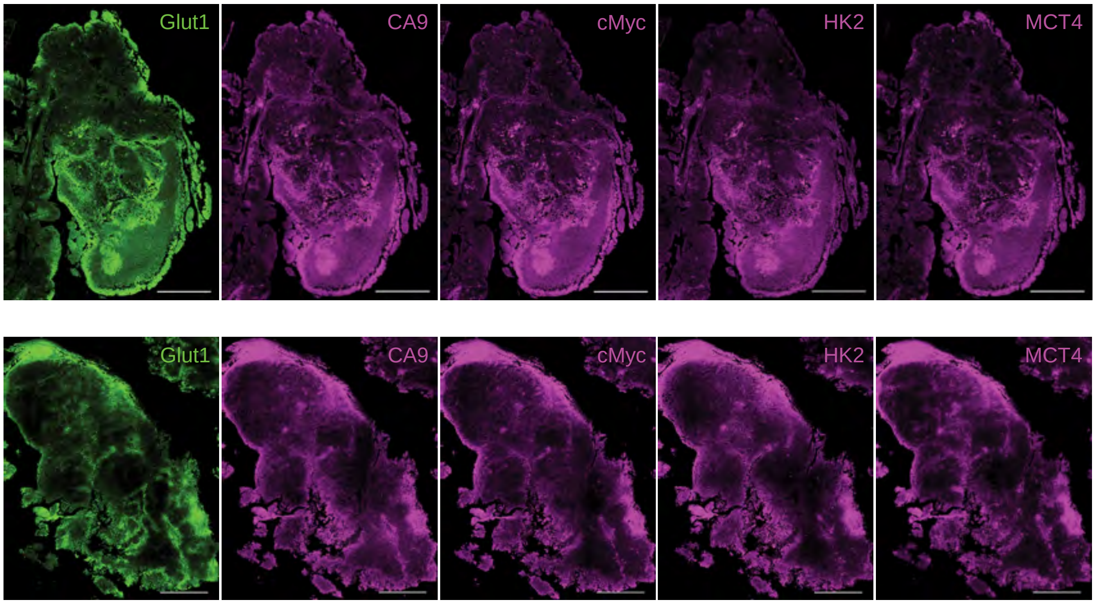

Figure S29. High correlation between multiple markers of glycolysis activation in primary and metastatic osteosarcoma lesions. A) Correlation of fluorescence intensity of markers of glycolysis (CA9, HK2, MCT4), an upstream regulator (cMYC) with GLUT1 along a ~3mm profile line drawn through the corresponding primary and metastatic lesions. The location and intensity of staining was highly correlated with GLUT1 for all markers. B) Fluorescent imaging of markers of glycolysis (CA9, HK2, MCT4) and an upstream regulator (cMYC) with GLUT1 along a ~1mm profile line drawn through the 143B lung lesions. The location and intensity of staining shows similarity to that of GLUT1 for all markers. Representative of n=3 mice.
