# Supplementary material for: Osteosarcoma tumors maintain intra-tumoral transcriptional heterogeneity during bone and lung colonization
Source: BMC Biol. 2023 Apr 27;21:98. doi: 10.1186/s12915-023-01593-3 (PMC10142502; doi:10.1186/s12915-023-01593-3)
Supplement: Supplementary file 15 — Additional file 15: Figure S30. Primary tumors demonstrate heterogeneity in GLUT1 staining. [file 12915_2023_1593_MOESM15_ESM.pdf]

Figure S30

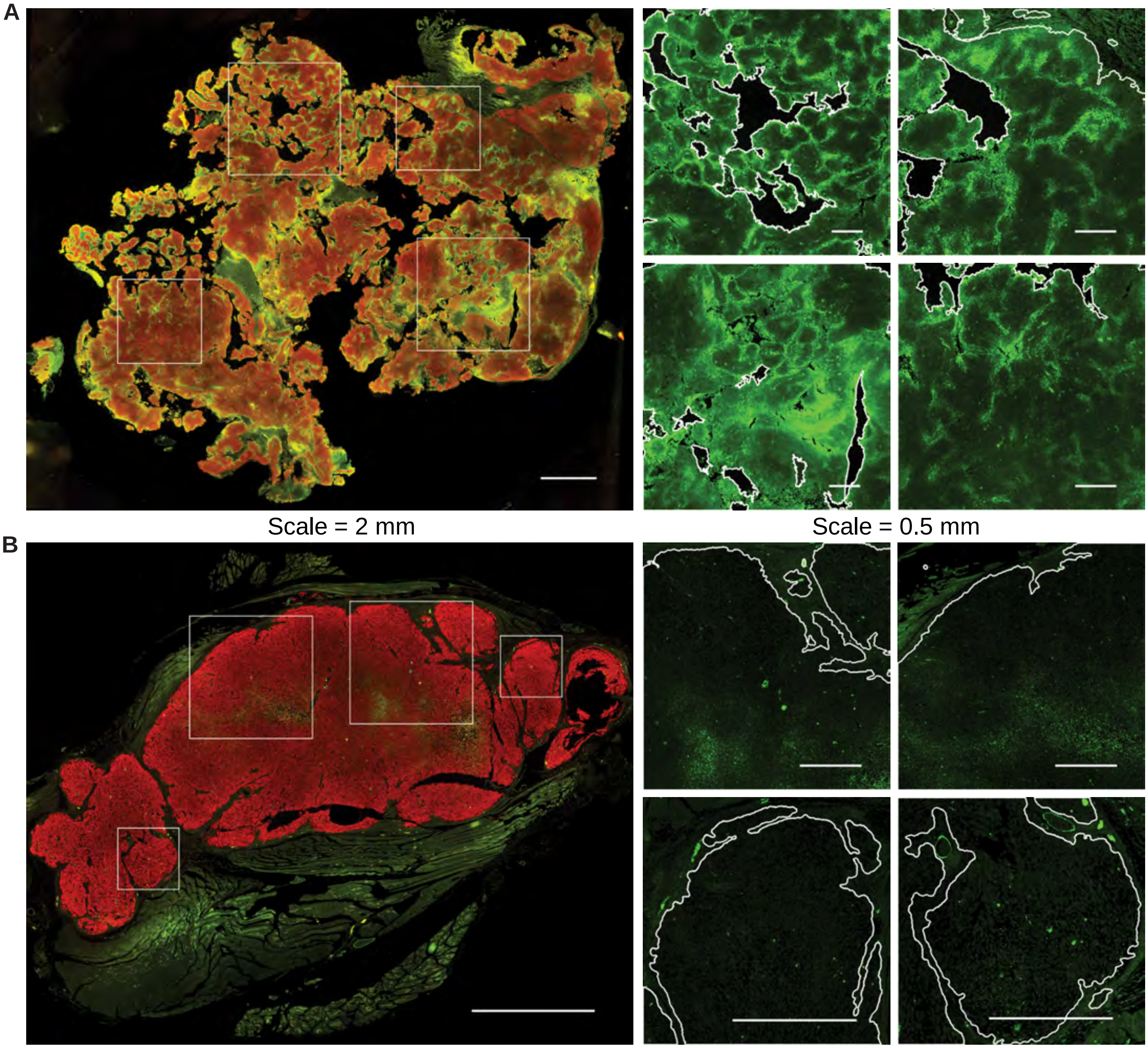

Figure S30. Primary tumors demonstrate heterogeneity in GLUT1 staining. A, B) Immunofluorescence staining of OS-17 and 143B primary tumors, respectively, for GLUT1 (green; a marker of glycolysis) and vimentin (red; marker to identify osteosarcoma cells). Magnified GLUT1 staining is shown for the boxed regions on the whole-section images. Lesion edges are indicated by white outlines in the magnified regions. Tumors showed a high degree of intra-tumor variation in GLUT1 staining intensity. Intensity ranged from strong to light in OS-17 and moderate to light in 143B, in which only some cells showed moderate expression of GLUT1.
