# Supplementary material for: Osteosarcoma tumors maintain intra-tumoral transcriptional heterogeneity during bone and lung colonization
Source: BMC Biol. 2023 Apr 27;21:98. doi: 10.1186/s12915-023-01593-3 (PMC10142502; doi:10.1186/s12915-023-01593-3)
Supplement: Supplementary file 16 — Additional file 16: Figure S31. Tumor cell cultures demonstrate heterogeneity in GLUT1 staining. [file 12915_2023_1593_MOESM16_ESM.pdf]

Figure S31

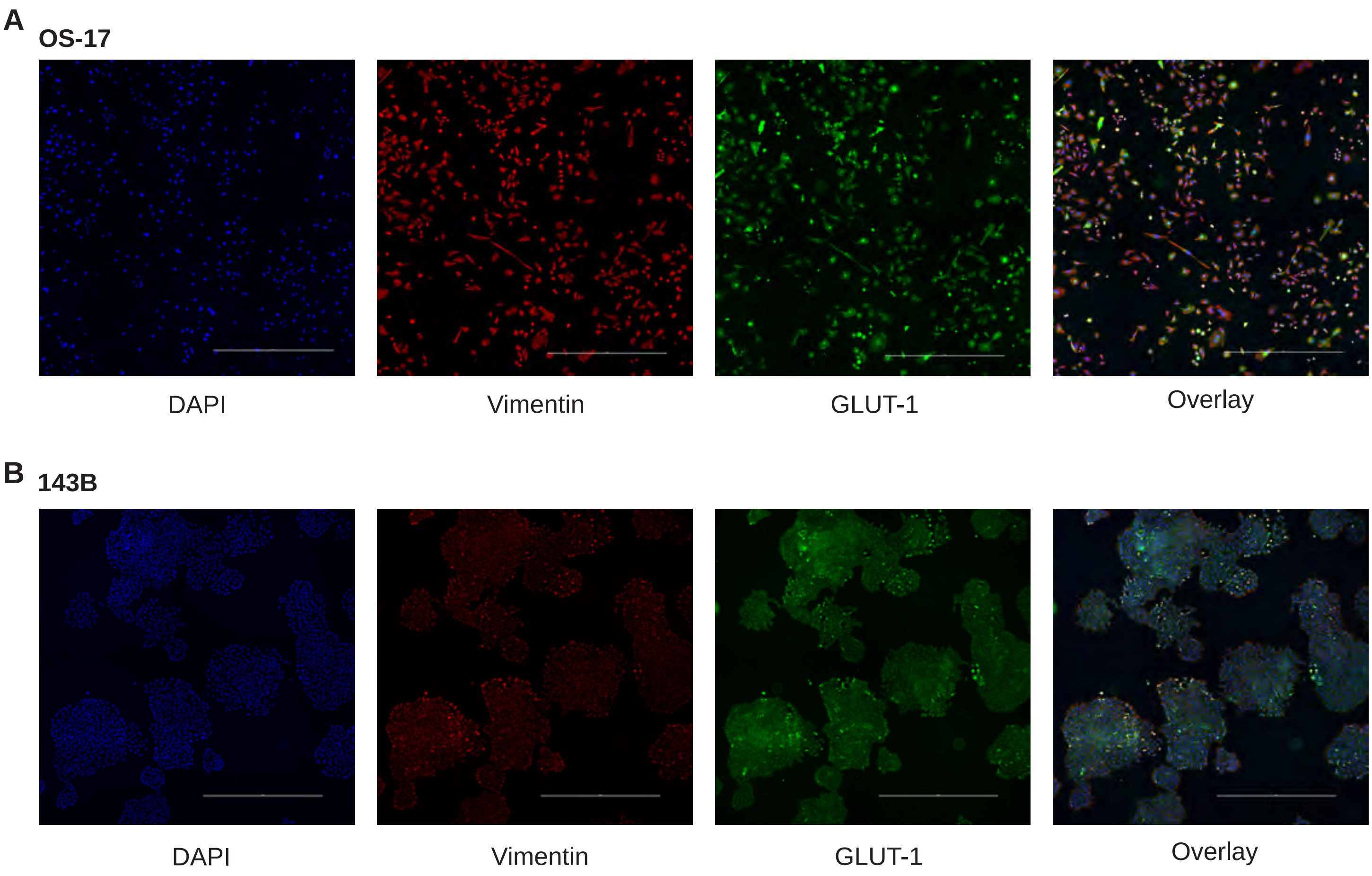

Figure S31. Tumor cell cultures demonstrate heterogeneity in GLUT1 staining. A, B) Immunofluorescence staining of OS-17 and 143B cell culture, respectively, for GLUT1 (green; a marker of glycolysis) and vimentin (red; marker to identify osteosarcoma cells) with scale bars of 1000 micrometers. Both cell culture models displayed strong to light expression of GLUT1.
