# Supplementary material for: Osteosarcoma tumors maintain intra-tumoral transcriptional heterogeneity during bone and lung colonization
Source: BMC Biol. 2023 Apr 27;21:98. doi: 10.1186/s12915-023-01593-3 (PMC10142502; doi:10.1186/s12915-023-01593-3)
Supplement: Supplementary file 17 — Additional file 17: Figure S32. Replicates demonstrate reproducible lineage tag enrichment profiles and consistent phenotypic profiles. [file 12915_2023_1593_MOESM17_ESM.pdf]

Figure S32

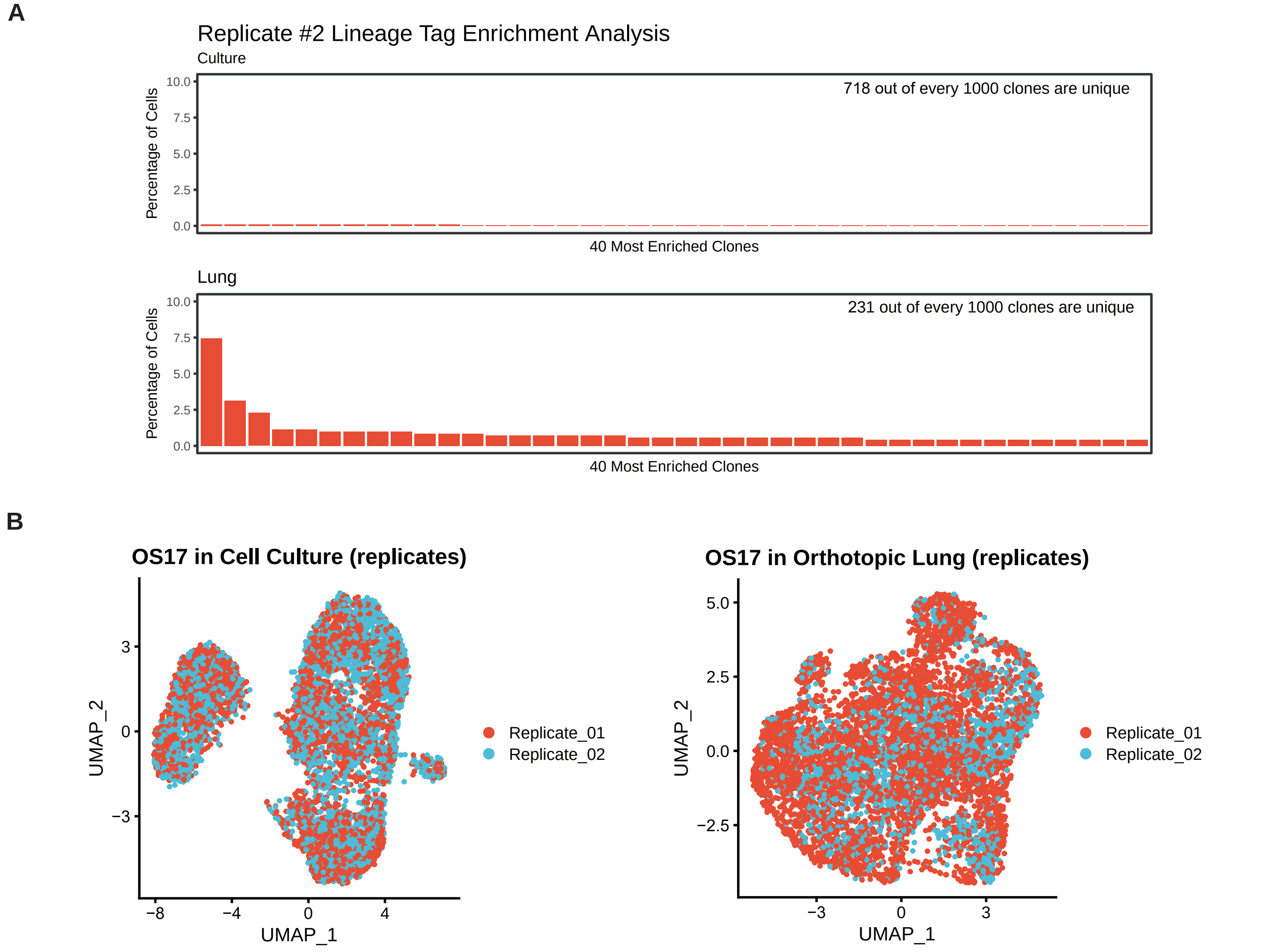

Figure S32. Replicates demonstrate reproducible lineage tag enrichment profiles and consistent phenotypic profiles. A) Frequency distribution of clones identified in each of the conditions generated using a biological replicate of the starting population (cells transduced with a separate batch of lentivirus library). B) Admixing of OS-17 cells from distinct, biological and technical replicates grown *in vitro* or as metastatic lesions suggests that intra-tumor transcriptional heterogeneity of osteosarcoma cells is not driven by individual tumor identity.
