# Supplementary material for: Osteosarcoma tumors maintain intra-tumoral transcriptional heterogeneity during bone and lung colonization
Source: BMC Biol. 2023 Apr 27;21:98. doi: 10.1186/s12915-023-01593-3 (PMC10142502; doi:10.1186/s12915-023-01593-3)
Supplement: Supplementary file 18 — Additional file 18: Figure S33. Contingency table displaying the number of enriched clones per cluster in culture, tibia, and lung. [file 12915_2023_1593_MOESM18_ESM.pdf]

Figure S33

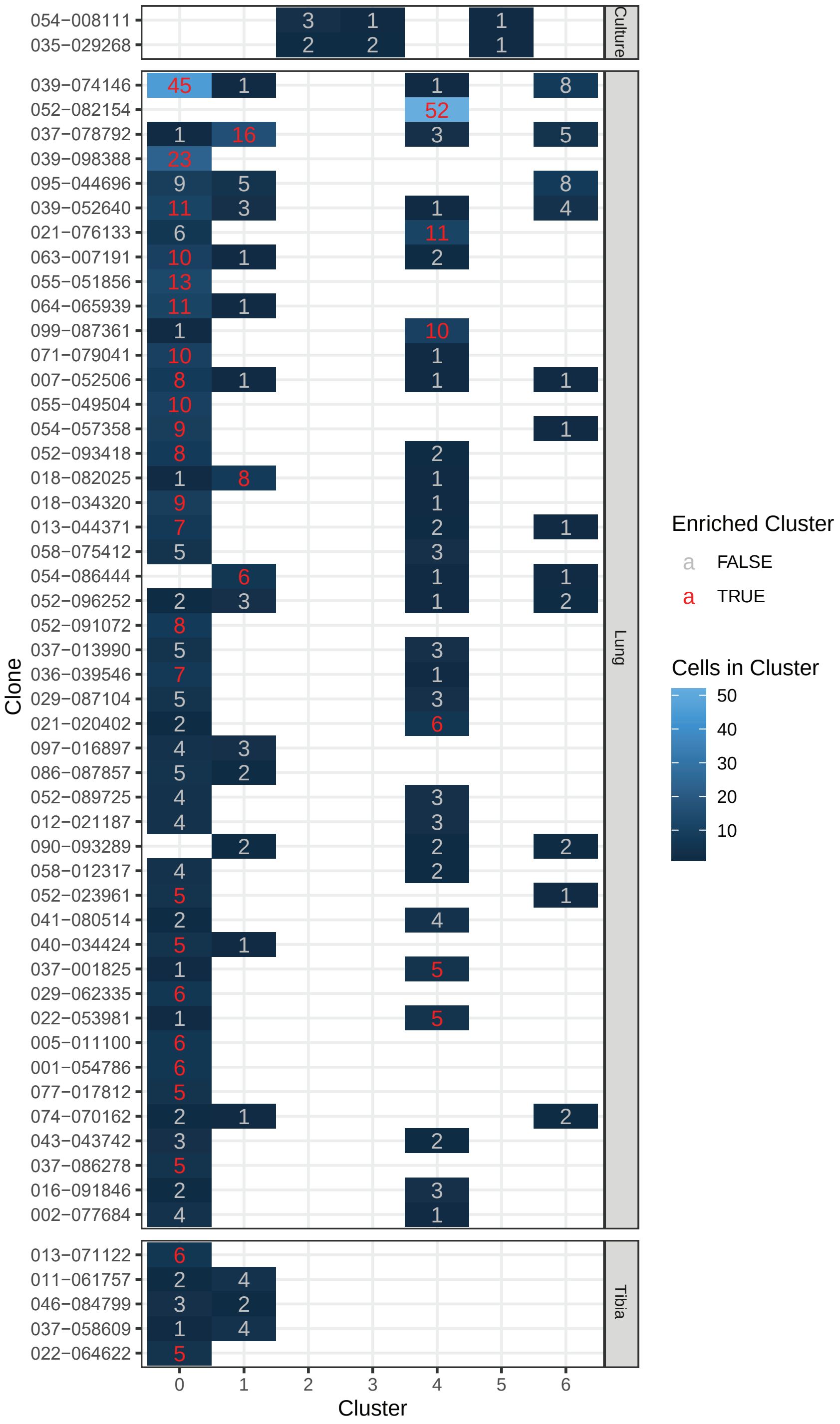

Figure S33. Contingency table displaying the number of enriched clones per cluster in culture, tibia, and lung. Numbers display how many cells from each clone are in a particular cluster from OS-17 tumors in the tibia or lung. Significant enrichment within one clone is indicated in red text, as calculated permutation analysis. The color of each box correlates to the total cells in each cluster.
